# A bispecific IgG format containing four independent antigen binding sites

**DOI:** 10.1038/s41598-020-58150-z

**Published:** 2020-01-31

**Authors:** Anne Ljungars, Torbjörn Schiött, Ulrika Mattson, Jessica Steppa, Björn Hambe, Monika Semmrich, Mats Ohlin, Ulla-Carin Tornberg, Mikael Mattsson

**Affiliations:** 1 BioInvent International AB, Lund, Sweden; 2 Department of Immunotechnology, Lund University, Lund, Sweden; 3 Present Address: SenzaGen AB, Lund, Sweden; 4 Present Address: Tetra Pak, Lund, Sweden; 5 Present Address: Hansa Medical AB, Lund, Sweden; 6 Present Address: Apotek Produktion och Laboratorier AB, Malmö, Sweden

**Keywords:** Biotechnology, Drug discovery, Immunology, Molecular biology

## Abstract

Bispecific antibodies come in many different formats, including the particularly interesting two-in-one antibodies, where one conventional IgG binds two different antigens. The IgG format allows these antibodies to mediate Fc-related functionality, and their wild-type structure ensures low immunogenicity and enables standard methods to be used for development. It is however difficult, time-consuming and costly to generate two-in-one antibodies. Herein we demonstrate a new approach to create a similar type of antibody by combining two different variable heavy (VH) domains in each Fab arm of an IgG, a tetra-VH IgG format. The VHs are used as building blocks, where one VH is placed at its usual position, and the second VH replaces the variable light (VL) domain in a conventional IgG. VH domains, binding several different types of antigens, were discovered and could be rearranged in any combination, offering a convenient “plug and play” format. The tetra-VH IgGs were found to be functionally tetravalent, binding two antigens on each arm of the IgG molecule simultaneously. This offers a new strategy to also create monospecific, tetravalent IgGs that, depending on antigen architecture and mode-of-action, may have enhanced efficacy compared to traditional bivalent antibodies.

## Introduction

Bispecific monoclonal antibodies have during the last decades emerged as a new growing class of antibody therapeutics^1–3^. While a normal, wild-type IgG is monospecific and typically binds one antigen, a bispecific antibody, by design, binds two structures simultaneously - either two different antigens, or two different epitopes on the same antigen. Bispecific antibodies can for example be used to recruit immune cells to tumors, to block two different pathways simultaneously, or to increase antibody specificity, resulting in a more effective therapy^2^ compared to conventional treatment with monospecific antibodies.

There are today numerous formats available for generation of bispecific antibodies^4,5^ and new ones are constantly being developed. The molecules differ in several aspects including size, symmetry, presence/absence of an Fc-domain, number of antigen binding sites, and number of antigens that can bind simultaneously. Formats constructed as whole IgGs share advantages with wild-type IgGs. This includes ability to bind Fc-receptors on effector cells to mediate antibody-dependent cellular cytotoxicity (ADCC) and antibody-dependent cellular phagocytosis (ADCP), or induction of complement dependent cytotoxicity (CDC). Typically, antibodies in an IgG format also show long half-lives due to their size and binding to FcRn.

One particularly interesting bispecific format, with a completely intact wild-type IgG structure, is the two-in-one antibody also called a dual action Fab (DAF), in which one variable region of an antibody binds two different antigens. This was first reported by Boström *et al*. who constructed an antibody binding to both HER2 and VEGF through mutagenesis of the HER2 specific antibody Herceptin^6^. This approach has since then been used to generate antibodies binding to additional antigen combinations such as HER3-EGFR^7^, VEGF-Angiopoietin 2^8^ or IL4-IL5^9^. It is, however, challenging to construct a DAF that binds two antigens with high affinity. In these antibodies the binding surfaces overlap and consequently only one antigen can bind each arm of the IgG at the time. This has partly been overcome in the DutaMab format where the binding site of the antibody is restricted to 3 of the 6 complementarity-determining regions (CDRs), making CDR H1, H3 and L2 binding one antigen and CDR L1, L3 and H2 binding a second antigen^4^. These DutaMabs can thus bind two different antigens, if allowed by antigen size and binding orientation, on each Fab arm simultaneously^10^. Another approach to construct a two-in-one type of antibody is to combine a variable heavy domain (VH) binding to one antigen with a variable light domain (VL) binding a second antigen^11^. All these strategies are however difficult, time-consuming and costly and methods for simple construction of the two-in-one antibody type of bispecifics are currently lacking.

The smallest antibody fragment commonly found to have antigen binding capacity is the VH domain^12–14^, and in animals of the camelidae family^15^ and in sharks^16^ antibodies consisting of only heavy chains have been found. This finding has been used for discovery and development of single domain antibodies, called nanobodies, recently reviewed by Mir *et al*.^17^. However, instead of creating a small nanobody, we hypothesized that human wild-type VHs can be used to create a tetravalent, whole IgG.

**Figure 1:**
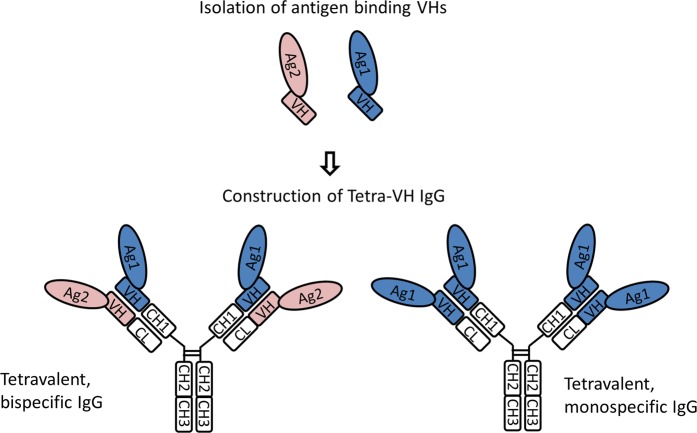
Schematic outline for construction of tetra-VH IgGs. Binding VHs can be isolated from VHs, scFvs, Fabs, or IgGs. These VHs are used as building blocks to create either bispecific or monospecific tetra-VH IgGs.

In this study we generate tetra-VH IgGs, a novel bispecific, tetravalent, two-in-one type of antibody (Fig. 1) using VH domains as building blocks. After isolation of binding scFv, through phage display, and identification of binding VH domains, one VH is linked to the constant heavy domain 1 (CH1) of the heavy chain, (normal position), whereas another VH is linked to the constant light domain (CL) replacing VL of a wild-type IgG. The selected VH domains typically bind different epitopes on one antigen or two different antigens. As the two VH domains bind independent of each other, tetravalent antibodies are obtained. This “plug and play” format shares several of the advantages of a normal wild-type IgG, and comprises a unique possibility to create tetravalent IgGs as an alternative format for drug development.

## Results

### Isolation of monospecific antibody fragments against CD40, OX40 and 4-1BB

**Figure 2:**
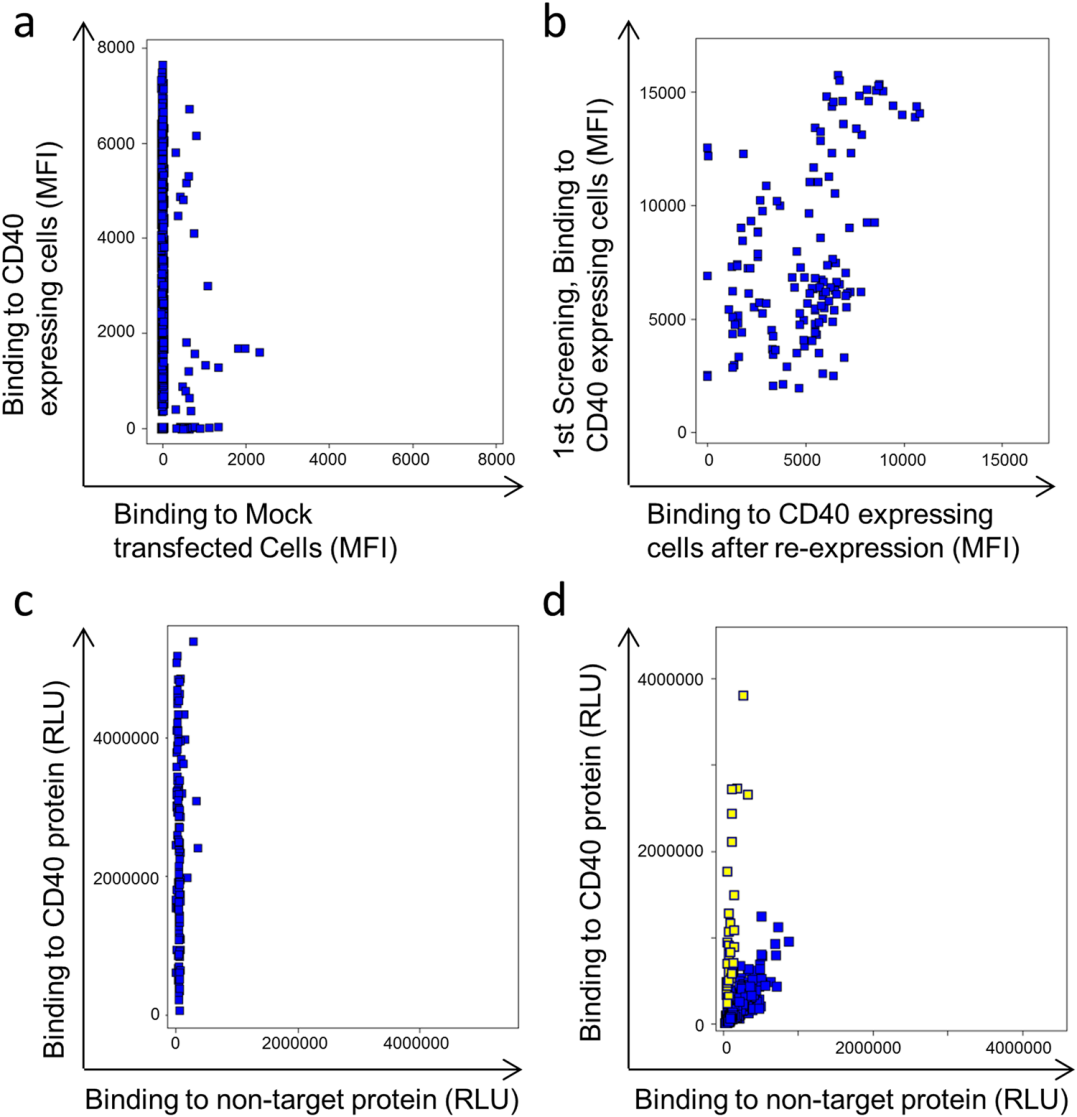
Binding-analysis of monospecific antibody fragments. (**a**) Screening in FMAT of individual soluble scFv showing the binding to CD40 expressing cells versus binding to mock transfected cells. (**b**) Binding of unique clones to CD40 expressing cells in the first screening versus their binding after re-expression. (**c**) Binding of clones in ELISA to coated recombinant CD40 and a non-related protein carrying the same tag as the target (non-target). (**d**) Screening of VH-VLD Fab clones in ELISA for binding to target versus non-target protein. Yellow-marked clones were selected as binders and used for Sanger sequencing to identify unique sequences.

The first step to generate bispecific tetravalent IgG molecules was to isolate monospecific antibody fragments against three model antigens, CD40, OX40 and 4-1BB. Target-specific cell binding scFv (single chain fragment variable) were isolated from our phage display library n-CoDeR^18^ (Figs. 2a, S1a). Unique clones, identified through Sanger sequencing, were re-produced and target binding confirmed using both transfected cells (Figs. 2b, S1b) and recombinant proteins (Figs. 2c, S1c). Altogether, around 100 clones/target were isolated for downstream evaluation.

### Isolation of monospecific VH domains to CD40, OX40 and 4-1BB

**Figure 3:**
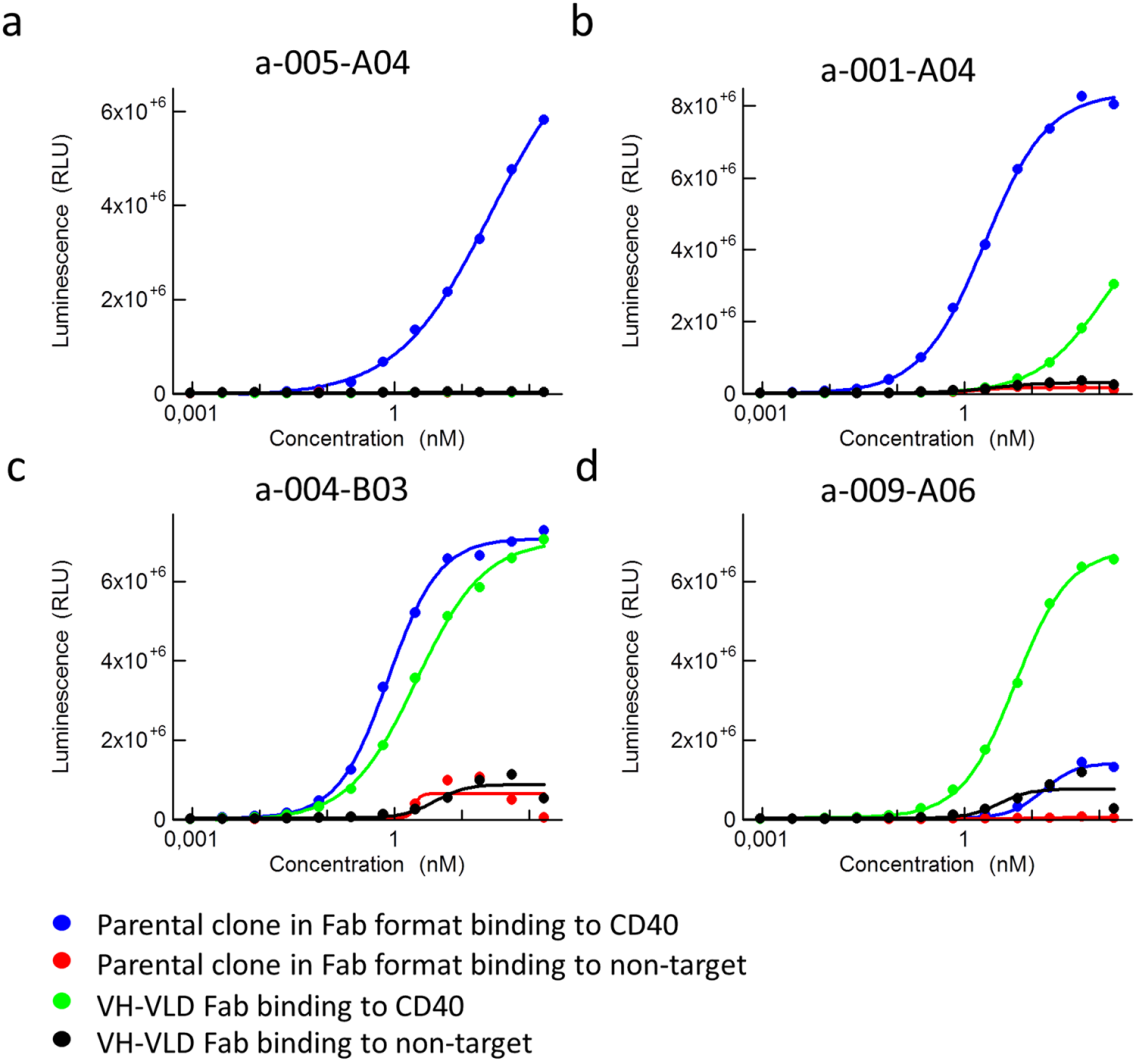
Dose response ELISA showing the binding of anti-CD40 VH-VLD Fab clones, compared to their corresponding parental clones, in a Fab format, to the CD40 target protein and a non-target protein. (**a**) Binding of clone a-005-A04. (**b**) Binding of clone a-001-A04. (**c**) Binding of clone a-004-B03. (**d**) Binding of clone a-009-A06.

To identify clones that bound with their VH alone, irrespectively of the VL, sub-libraries of the isolated monospecific scFv were made. VH genes from all unique scFv, binding to one target, were pooled and combined with a VL dummy (VLD), in a vector used for production of Fab (fragment antigen binding), here called VH-VLD Fabs. Individual VH-VLD Fabs were screened for binding to recombinant proteins in ELISA (Figs. 2d and S1d). Binding, unique VH-VLD Fabs were produced, purified and analyzed in a dose response ELISA to confirm target binding (Figs. 3 and S2). Binding of the parental clone in a Fab format, containing the original VL, was included as control. Different binding patterns were seen including: binding of parental Fab with no binding of the VH-VLD Fab (Fig. 3a), stronger binding of parental Fab compared to VH-VLD Fab (Fig. 3b), similar binding of VH-VLD Fab and parental Fab (Fig. 3c), and stronger binding of VH-VLD Fab than the parental Fab (Fig. 3d). The latter showed that the VL of some selected scFv may even inhibit its VH partner from binding.

As an alternative to VH-VLD screening, VHs can be expressed and analyzed for binding without the light chain. This was tested for a few clones known to contain a VH that is suitable for construction of tetra-VH IgGs. Purified VHs bound similar to the target antigen as scFv for most clones (Fig. S3). The VH-VLD screening can thus be replaced with a “VH only” screening for identification of VHs.

### Construction and binding analysis of bispecific tetra-VH IgG antibodies

VH domains from isolated target specific VH-VLD clones were used as building blocks and combined in an IgG format to create bispecific tetra-VH IgG antibodies. Genes encoding two different VHs were fused to either the gene encoding CH1, the normal position of VH, or to the gene encoding CL, replacing VL, in vectors encoding the constant domains of a human IgG1 antibody. The constructs were used for production of IgG molecules, called tetra-VH IgGs (Fig. 1). Generated tetra-VH IgGs were purified according to standard procedures on protein A followed by preparative size exclusion chromatography, SEC, to remove any free VH-containing light chains. The amount of co-purified monomeric or dimeric VH containing light chains varied substantially between different antibodies (Fig. S4a). This was likely due their different sequences and thereby different affinity for protein A. After removal of any free light chains the antibodies were analyzed with CE-SDS (Capillary electrophoresis sodium dodecyl sulfate) and showed the expected size and composition (Fig. S4b).

**Figure 4:**
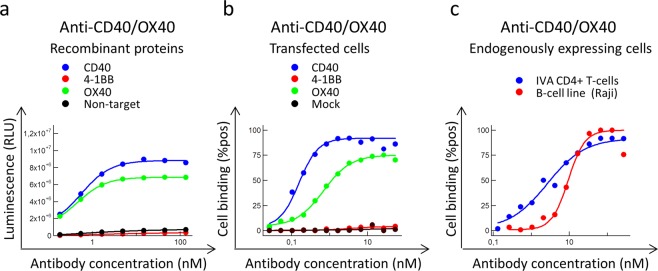
Binding-analysis of a CD40/OX40 specific tetra-VH IgG. The antibody was evaluated for (**a**) binding to recombinant proteins in ELISA and for binding to (**b**) transfected cells or (**c**) endogenously expressing cells in flow cytometry. *In vitro* activated (IVA) CD4+ T-cells express OX40 and 4-1BB and the B-cell line Raji express CD40.

**Figure 5:**
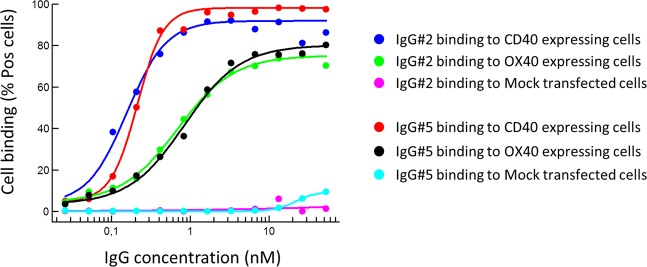
Binding-analysis of bispecific tetra-VH IgGs to overexpressing cells in flow cytometry. The two VHs, binding CD40 and OX40 respectively, were either linked to CH1 or CL in the antibody. In IgG #2 the CD40 specific VH is linked to CL and the OX40 specific VH is linked to CH1. In IgG #5, it is the other way around, the OX40 specific VH is linked to CL and the CD40 specific VH is linked to CH1.

To evaluate the functionality, with respect to antigen binding, antibodies were titrated for binding, including commercial antibodies against CD40, 4-1BB and OX40 that confirmed target expressions on included cells (Fig. S5). The tetra-VH antibodies were bispecific and bound two antigens, either CD40/OX40, 4-1BB/CD40 or OX40/4-1BB, as recombinant proteins (Figs. 4a and S6a) or expressed on the cell surface (Figs. 4b,c and S6b,c). In total, around 7 percent of the generated monospecific scFv, mean of all three targets, bound in the Fab VH-VLD format and several (40%) of these VHs could be produced and retained binding as tetra-VH IgGs (Supplementary Table 1). This demonstrated that monospecific VHs could be combined in various combinations, offering a novel approach to generate bispecific antibodies. Importantly, the binding VH domains could be placed at both the CL- and CH1-position without affecting the binding affinity and specificity of the antibody (Fig. 5).

### Stability of bispecific tetra-VH IgG antibodies

To examine the stability of the generated tetra-VH IgGs, some purified IgG preparations were incubated in 50% human serum at +37 °C for up to 7 days followed by binding analysis to coated antigen in ELISA. In addition, purified IgG preparations were stored at +4 °C for 3 years followed by repeated binding analysis to recombinant proteins and size exclusion chromatography (SEC) analysis. The tetra-VH IgGs bound similar to the antigen after 7 days incubation at +37 °C in 50% human serum (Fig. S7a). After long term storage, the tetra-VH IgGs bound with similar EC50 values in ELISA and showed no aggregation in SEC (Supplementary Table 2 and Fig. S7b). This demonstrated that the tetra-VH IgGs were very stable.

The thermal stability of the tetra-VH IgGs was evaluated with nano differential scanning fluorimetry (nano-DSF), with IgGs containing a variable light dummy chain included for comparison. The tetra-VH IgGs showed similar variation in thermal stability as conventional IgGs (Fig. S7c).

### *In vitro* testing of bispecific tetra-VH IgG antibodies

**Figure 6:**
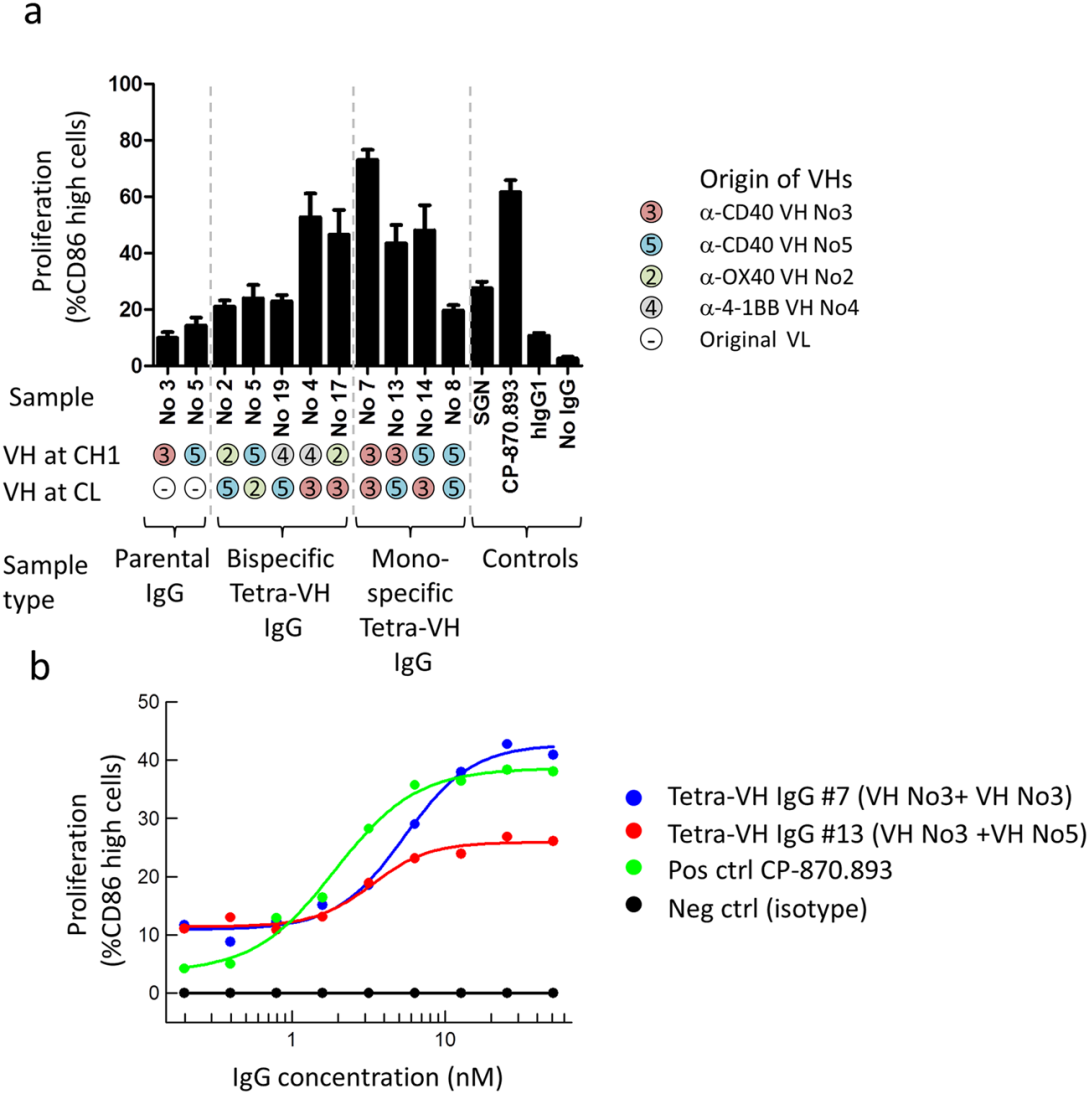
Evaluation of antibodies effect on B-cells proliferation. (**a**) Wild-type parental IgGs, tetra-VH IgGs and control antibodies, were cross-linked and analyzed for induction of B-cell proliferation on B-cells from healthy donors. Proliferation was measured as the percent of live CD19+ cells that were CD86 high. Samples were run as duplicates and data from 2–4 different donors were plotted as mean with SEM using GraphPad Prism. (**b**) Dose response analysis of antibodies effect on B-cell proliferation showing the mean, after subtraction of values for the isotype control, from 2 different experiments using B-cells from 4 different donors.

The functionality of generated anti-CD40 tetra-VH IgGs, with respect to agonistic activity, was analyzed in a B-cell proliferation assay. VHs from two anti-CD40 antibodies, including one with agonistic activity (denoted VH No 3), were combined and analyzed as: wild-type monospecific IgGs, bispecific tetra-VH IgGs or monospecific tetra-VH IgGs. In the tetra-VH IgG format the two anti-CD40 VHs were combined with each other, alternatively with VHs targeting OX40 or 4-1BB representing, in this context, a non-binding VH as neither OX40 nor 4-1BB are expressed on B-cells. Two known agonistic anti-CD40 antibodies, a human IgG2, CP-870.893 (Pfizer/VLST), and a humanized IgG1, SGN (also called Dacetuzumab or huS2C6 from Seattle Genetics) were included as positive controls. To mimic the situation *in vivo*, where antibodies are commonly cross-linked through Fc-receptor binding, an anti-human Fc specific F(ab’)2 antibody was included for crosslinking. No effect of the tetra-VH IgGs or the positive control antibody SGN was seen without crosslinking (Fig. S8). In contrast, the positive control antibody CP-870.893, known to be agonistic independent of crosslinking^19^, induced B-cell proliferation without crosslinking. However, after crosslinking, tetra-VH IgGs containing the agonistic VH, both in a monospecific or in a bispecific format, induced B-cell proliferation similar to the positive control antibody CP-870.839 and more than the positive control SGN (Fig. 6a). Importantly, this showed that the functional activity (here agonistic activity) of the VH domain was maintained in the tetra-VH IgG format regardless of the position and VH-partner. The wild-type monospecific IgG No3 induced no proliferation, in contrast to tetra-VH IgG containing this VH. This was most likely due to very weak binding of the wild-type IgG. This binding was significantly improved in the tetra-VH IgG format when the parental VL was removed. Dosing of a few of the most potent antibodies, demonstrated that combining two agonistic VHs in each arm of an IgG, induced about twice as much proliferation as an antibody containing only one agonistic VH, in a cross-linked set-up (Fig. 6b).

### Analysis of simultaneous binding of two antigens to tetra-VH IgGs

**Figure 7:**
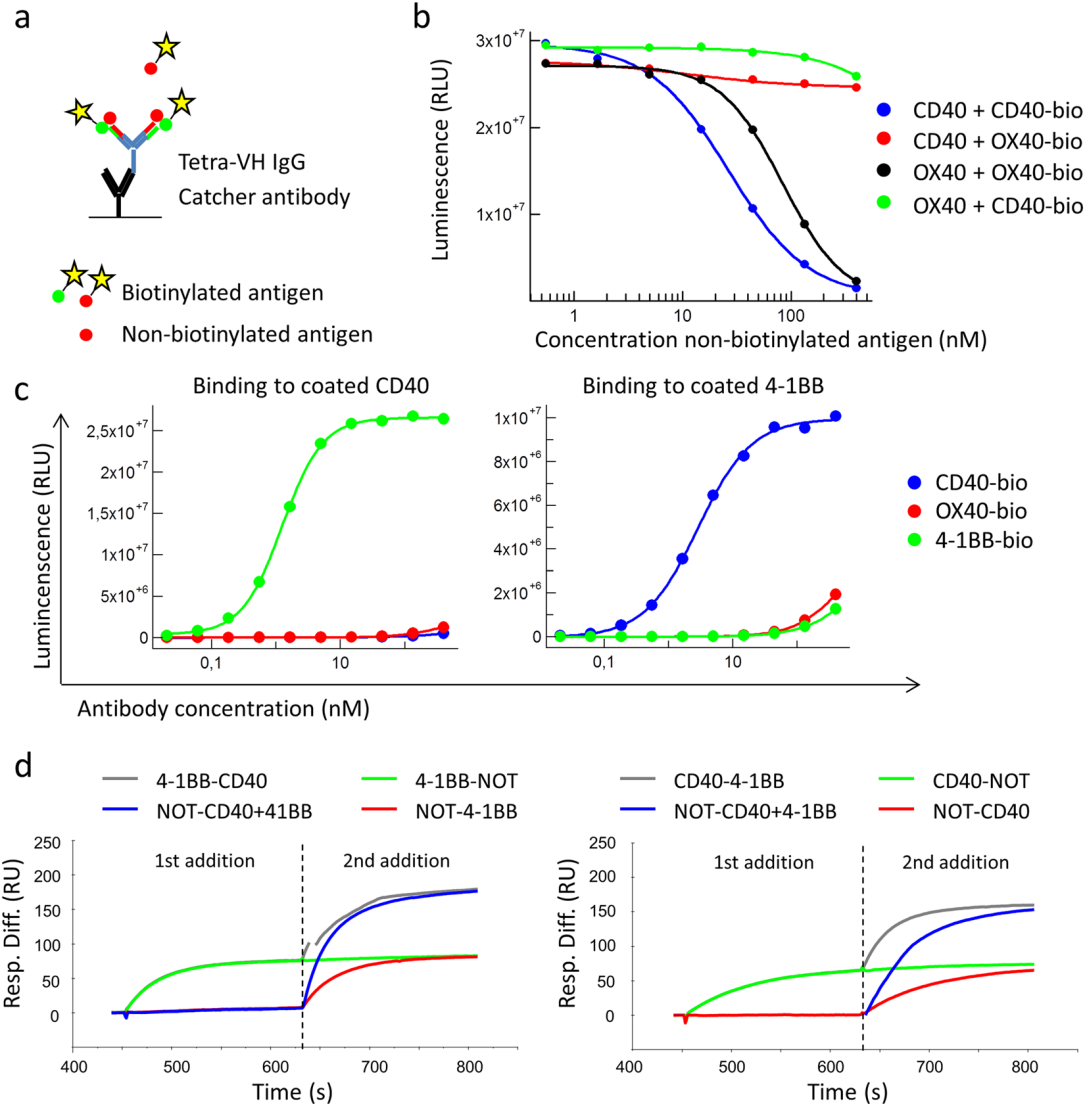
Binding-analysis of bispecific tetra-VH IgGs and Fabs capability to bind two different antigens simultaneously. (**a**) Schematic outline of the ELISA set-up. Tetra-VH IgGs were captured on a coated anti-human-Fc specific antibody followed by addition of a non-biotinylated antingen. Binding of the same or a second antigen, in a biotinylated version, was then detected using HRP-labeled Streptavidin and a luminescent substrate. (**b**) ELISA analysis of an anti-CD40/OX40 tetra-VH IgG for binding to biotinylated CD40 or OX40 after blocking with non-biotinylated CD40 or OX40. The assay was run as described in A. (**c**) Bridging ELISA. An anti-CD40/4-1BB Fab was analyzed for binding to coated CD40 respectively 4-1BB followed by addition of biotinylated CD40, OX40 or 4-1BB antigen. Binding of biotinylated antigens was detected using Streptavidin-HRP and a luminecent substrate. (**d**) Binding-analysis of CD40 and 41BB to an anti-41BB/CD40 tetra-VH IgG in Biacore. Antibodies were captured on an immobilize catcher antibody followed by addition of 800 nM of the first antigen to achieve binding saturation. The second antigen was then added at 800 nM, diluted in 800 nM of the first antigen, to avoid signal loss due to dissociation of the first antigen.

To analyze if the constructed bispecific tetra-VH IgGs were tetravalent and thus could bind two antigens simultaneously on each Fab arm, the antibodies were analyzed in ELISA and Biacore. In ELISA, non-biotinylated antigen was added at different concentrations to captured tetra-VH IgGs followed by addition and detection of a fixed amount of biotinylated antigen (Fig. 7a). For a CD40/OX40 bispecific antibody it was seen that CD40 inhibits the binding of biotinylated CD40 and OX40 inhibits the binding of biotinylated OX40 in a dose dependent manner, as expected. However, CD40 showed no inhibition of biotinylated OX40 and vice versa (Fig. 7b). The same pattern was seen for antibodies targeting 4-1BB/CD40 or OX40/4-1BB (Fig. S9). This indicated a tetravalent binding as the first antigen was unable to affect the binding of the second antigen to the tetra-VH IgG, and vice versa. In addition, a bridging ELISA with a CD40/41BB bispecific VH-VH Fab was run. This showed that the VH-VH Fab can bind both coated CD40 antigen and biotinylated 4-1BB in solution simultaneously or vice versa (Fig. 7c). This demonstrated that each Fab arm of the tetra-VH IgG can bind two antigens simultaneously.

The tetravalent binding was further confirmed in Biacore. Tetra-VH IgGs were captured on an immobilized catcher antibody followed by sequential antigen additions. After binding saturation with the first antigen, addition of the second antigen resulted in increased binding. The same amount of the second antigen bound regardless if the first antigen was added or not. No added signal was seen after addition of a non-target protein or repetitive addition of the same antigen (Fig. 7d). All antigen combinations, CD40/OX40, 4-1BB/CD40 or OX40/4-1BB, showed the same result (Figs. 7d and S10). Importantly, this clearly demonstrates that the generated tetra-VH IgGs are tetravalent and can bind two antigens simultaneously on each Fab arm.

### Identification of VHs binding to additional targets

CD40, OX40 and 4-1BB all belong to the tumor necrosis factor receptor superfamily. To confirm a broad applicability of the tetra-VH approach, VHs binding to additional antigens were analyzed. A VH binding to the cytokine MCP-1 was identified from a previously identified MCP-1 specific scFv. This anti-MCP-1-VH could be combined with any of the previously generated VH-specificities (CD40, OX40, 4-1BB) (Fig. S11a). In addition, VHs binding to CD72, which belongs to another type of cell surface receptors, the c-type lectin family, were also isolated (Fig. S11b). This validated that VH domains binding various types of antigens can be found.

## Discussion

In this study we generate a new type of bispecific antibody, a tetra-VH IgG, through the combination of two VH domains in each arm of an IgG molecule, one linked to CL, (replacing the VL domain) and the other linked to CH1 (the normal position of VH). The different VH domains can be combined in any combination, offering a convenient “plug and play” format to generate a two-in-one type of IgG antibody with the specificity of interest. The IgG format with an unmodified Fc allows the antibodies to be used for ADCC, ADCP or CDC in contrast to several other bispecific formats^4^, where building blocks of binding domains, such as scFv or Fabs, are combined and linked together without any Fc-domain.

We show that VH domains binding different types of antigens can be easily isolated after initial selection using a scFv-phage display library and used as building blocks for construction of tetra-VH IgG antibodies. Other approaches to create two-in-one type of antibodies require either mutagenesis of an already discovered antibody^6–9,20^ to enable a second antigen to bind with high affinity within the binding site, the use of two different binding sites within one IgG molecule^10^ or the combination of a VH binding to one antigen with a VL binding a second antigen^11^. The latter shares the advantage with our strategy, that building blocks are generated, which can be reused in different combinations. It is however often challenging to find binding VL domains as the VL of an IgG is, at least those carrying only natural diversity, much less variable than the VH, due to how antibody variability is created during B-cell development^21^. This limitation was avoided by the use of VH domains only in the creation of tetra-VH IgGs.

In this study, standard methods, used for production of wild-type IgGs, were applied also on the tetra-VH IgGs, with the only exception that a preparative SEC was run to remove unbound VH containing light chains after purification on protein A. Free light chains are commonly secreted by cells, but are not captured by protein A. In this case, however, the presence of a VH domain in the light chain results in co-purification of light chains. The ability of VH-domains to bind to protein A depends on the amino acid sequence, where VH-genes from the IGHV3 family, commonly used both in libraries (such as n-CoDeR) and in nature, have the highest affinity for protein A^22,23^. The co-purification of the VH-containing light chains can presumably be avoided by using a different resin in the purification step^24^, possibly in combination with the introduction of a mutation in the protein A binding structure of CDRH2^25^. The stability of the tetra-VH IgGs, over 3-years’ time, with respect to aggregation, binding affinity and specificity, was very good. In addition, the tetra-VH IgGs retained their binding properties after one week of incubation at +37 °C in 50% human serum, which further demonstrates their stability and wild-type like properties. The wild-type IgG architecture of the molecules most likely reduces the need for a separate method-development to process these antibodies, which is often associated with the development of other types of bispecific antibodies^26,27^, although the final developability^28^ (and immunogenicity) remains to be assessed.

The limiting step in this study was the isolation of the antibody fragments capable of binding with the VH domain alone. Once VHs were isolated many of them could be used to create tetra-VH IgGs. To improve this, although not attempted in in this study, the concept can be further developed by creating a library of phages displaying VHs only^12–14^, which can be used for direct isolation of antigen-specific VH building blocks.

In summary, VH domains binding several antigens of various types were isolated and successfully used in a tetra-VH IgG format, offering a novel, fast, and easy strategy for development of tetravalent mono- or bi-specific antibodies. As both VH domains in one Fab arm retain their binding properties, a tetravalent format, where four antigen molecules can bind simultaneously to one tetra-VH IgG, was created. This offers possibilities beyond the traditional bispecific antibody formats, as it also enables a new type of monospecific, tetravalent antibodies to be developed, constructs that may show enhanced binding and functional properties.

## Material and Methods

### Detailed methods are provided as supplementary material and methods

Ethical approval for human cells was obtained by the Ethics Committee of Skåne University Hospital and informed consent was provided in accordance with the declaration of Helsinki. All experiments were performed in accordance with the local guidelines and regulations.

### Isolation of monospecific antibody fragments

CD40-, OX40-, and 4-1BB- specific scFv displayed on phages were obtained from the n-CoDeR scFv library^18^, using a combination of recombinant proteins (in-house produced) and target-expressing cells (in-house constructed) for selection. After 3 consecutive selection rounds, genes encoding the scFv were used for conversion to soluble scFv as described previously^29^.

### Isolation of monospecific VH binders

Individual scFv clones were analyzed for binding to target expressing and mock transfected CHO cells in FMAT (Flourometric Microvolume Assay Technology). Genes encoding VHs from all unique, binding scFv were amplified by PCR, pooled and ligated into a Fab expression vector (proprietary in-house developed) containing a VL dummy gene composed of the light chain variable gene IGLV1-47∗01 (accession no Z22189) rearranged to the light chain joining gene fragment IGLJ3*02 (accession no D87023). This vector was used to transform chemically competent E. coli Top10 and individual colonies were used for production of VH-VLD Fabs, followed by binding analysis to recombinant proteins in ELISA. Unique, binding VH-VLD Fabs were re-produced in E coli, purified from the periplasm using a Ni-NTA plate (His MultiTrap HP, 96 well, GE Healthcare cat no. 28-4009-89) and analyzed in a dose response ELISA. Binding in ELISA was detected using an AP-labeled anti-FLAG M2 antibody (Sigma Aldrich cat no. A9469) and a luminescent substrate (CDP Star Emerald II, Thermo Fisher cat no. T2216).

For expression of soluble VH domains, VH-encoding genes from clones binding in a VH-VLD Fab format were amplified by PCR from the scFvs, and ligated into a vector containing a 6xhis and 3xFLAG tag. Purification and ELISA binding analysis was performed as for VH-VLD Fabs above.

### Construction and stability analysis of tetra-VH IgG antibodies

The genes encoding the VH domains of VH-VLD binding Fabs were PCR amplified from the scFv vector and ligated into two expression vectors, a heavy and a light chain IgG vector, (proprietary, in-house developed) to enable production of tetra-VH human IgG antibodies. The heavy chain vector contains the gene encoding the antibody heavy chain IgG1 constant region and the light chain vector contains the gene encoding the antibody lambda light chain constant domain. One VH encoding gene is inserted in the vector containing the gene encoding the constant heavy chain (normal position), whereas the other VH is inserted in the vector containing a gene encoding the lambda light chain constant domain (CL), thereby replacing VL in the encoded product. The vectors were used for transient transfection of suspension-adapted HEK 293 EBNA (Thermo Fisher Scientific). IgGs were purified from the cell supernatants on Mabselect (GE Healthcare) followed by preparative size-exclusion chromatography (SEC) to remove unpaired VH containing light chains. After purification the antibodies were run in CE-SDS (Capillary electrophoresis sodium dodecyl sulfate) under reduced and non-reduced conditions using the HT Antibody Analysis 200 assay (LabChip GX II, Perkin Elmer). In addition, antibodies were analyzed by analytical SEC (Ultimate3000, Thermo Fisher) and this was repeated after long term storage at +4 °C, a gel filtration standard (at time point 3 years) (Biorad cat no. 1511901) or an in-house standard (time point 0 years) were included as controls. To evaluate the antibodies stability in in human serum, tetra-VH IgGs were diluted to 400 nM in 50% human serum (Sigma cat. no. H4522) in PBS and incubated for 0, 1, 3, 4, or 7 days at +37 °C before freezing. Frozen samples were thawed, diluted to 200 nM in ELISA block buffer (PBS (Invitrogen) with 0.05% Tween 20 and 0.45% fish gelatin (both from Sigma-Aldrich)), titrated 1:3 and analyzed for binding to recombinant proteins in ELISA as described below. The antibodies thermal stability was analyzed with nanoDSF (differential scanning fluorimetry) in Prometheus NT.48 from NanoTemper Technologies with a temperature ramp of 1 °C/min from 20 to 95 °C.

### Binding analysis of tetra-VH IgG antibodies

Purified tetra-VH IgG antibodies were analyzed for binding to recombinant proteins in ELISA and to transfected and endogenously expressing cells in flow cytometry. Binding in ELISA was detected using a horseradish peroxidase (HRP) conjugated anti-human-Fc antibody (Jackson ImmunoResearch cat no. 709-036-098), followed by a luminescent substrate (SuperSignal ELISA Pico Chemiluminescent Substrate, Thermo Fisher, cat no. 37070). In flow cytometry, binding was detected with anti-human F(ab)’2-APC (Jackson ImmunoResearch, cat no. 109-136-098).

### B-cell proliferation assay

Buffy coats from healthy donors (Lund University Hospital) were collected and B-cells, purified from buffy coats using a pan B-cell isolation kit (Miltenyi, cat no. 130-091-151), were incubated with 50 nM of antibodies with or without crosslinking. After 5 days incubation at +37 °C, 8% CO2, cells were stained with anti-CD19-BV421 (BD Biosciences, cat no. 562440), anti-CD86-APC (BD Biosciences, cat no. 555660) and a live dead marker (Thermo Fisher, propidium iodide, alternatively eBioScience, cat no. 65-0865-14). The fraction of live, CD19+ and CD86 high cells was analyzed by flow cytometry (FACS Verse, BD Biosciences).

### ELISA analysis of tetra-VH IgGs for evaluation of valency

The tetra-VH IgGs ability to bind two antigens simultaneously was assessed in ELISA, schematically outlined in Fig. 7a. Non-biotinylated antigen, diluted to 400 nM, was titrated 1:3 and added to captured tetra-VH IgGs followed by biotinylated antigen addition at a fixed concentration resulting in a high, but titratable, signal. After washing, bound biotinylated antigen was detected using HRP-labeled streptavidin (Jackson ImmunoResearch, cat no. 016-030-084) and a luminescent substrate (SuperSignal ELISA Pico Chemiluminescent Substrate).

To further investigate if each arm of the tetra-VH IgGs could bind two antigens simultaneously, a bridging ELISA was performed. VH-VH Fabs (produced as described for VH-VLD Fabs above) were left to bind the first coated antigen and after washing the second antigen was added at different concentrations in a biotinylated version. Thereafter, bound biotinylated antigen was detected using HRP-labeled streptavidin (Jackson ImmunoResearch, cat no. 016-030-084) and a luminescent substrate (SuperSignal ELISA Pico Chemiluminescent Substrate). A non-target protein and the coated antigen added in a biotinylated form were included as controls.

### Biacore analysis of tetra-VH IgG antibodies for evaluation of valency

A CM5 chip (GE Healthcare) was immobilized with anti-human IgG, 25 µg/ml, (GE Healthcare, cat no. BR 1008-39). IgG was added at 10 nM, 10 μl/min, for 3 min followed by the first antigen addition, 800 nM, 30 µl/min, for 3 min followed by the second antigen addition (diluted in 800 nM of the first antigen to avoid signal loss due to dissociation of the first antigen), 800 nM, 30 µl/min, for 3 min. The surface was regenerated with 10 mM glycine, pH 1.5, between each cycle.

### Generation of VHs against additional antigens

The VH-encoding gene from one MCP-1 binding clone (generated previously, unpublished) was amplified by PCR, ligated into an expression vector containing a 6xhis and 3xFLAG tag, and used for production of a soluble VH-VLD Fab. Purification and ELISA binding analysis was performed as described above. The anti-MCP-1 binding VH domain was combined with VH domains binding to CD40, OX40 and 4-1BB respectively, in a tetra-VH IgG format and binding was confirmed as describe above.

For generation of VH binding clones against CD72, VH-encoding genes from CD72 binding scFv clones^30^, were amplified by PCR, ligated into an expression vector containing a 6xhis and 3xFLAG tag (no dummy VL), and screened for binding to CD72 and a non-target protein in ELISA as described for VH-VLD Fabs. Unique clones, identified through Sanger sequencing, were used for production, purification, and ELISA binding analysis as described for the VH-VLD Fabs above.

## Supplementary information

Supplementary Information.
