# Supplementary material for: A bispecific IgG format containing four independent antigen binding sites
Source: Sci Rep. 2020 Jan 31;10:1546. doi: 10.1038/s41598-020-58150-z (PMC6994471; doi:10.1038/s41598-020-58150-z)
Supplement: Supplementary file 1 — Supplementary Information. [file 41598_2020_58150_MOESM1_ESM.pdf]

Supplementary Information

A bispecific IgG format containing four independent antigen binding sites

Anne Ljungars[1,2], Torbjörn Schiött[1], Ulrika Mattson[1,3], Jessica Steppa[1,4], Björn Hambe[1,5], Monika Semmrich[1], Mats Ohlin[2], Ulla-Carin Tornberg[1†*], Mikael Mattsson[1,6†]

[1]BioInvent International AB, Lund, Sweden, [2]Department of Immunotechnology, Lund University, Lund, Sweden,
[3] Current affiliation: SenzaGen AB, Lund, Sweden
[4] Current affiliation: Tetra Pak, Lund, Sweden
[5] Current affiliation: Hansa Medical AB, Lund, Sweden
[6] Current affiliation: Apotek Produktion och Laboratorier AB, Malmö, Sweden

† These authors contributed equally to this work

*** Correspondence: Ulla-Carin.Tornberg@bioinvent.com**

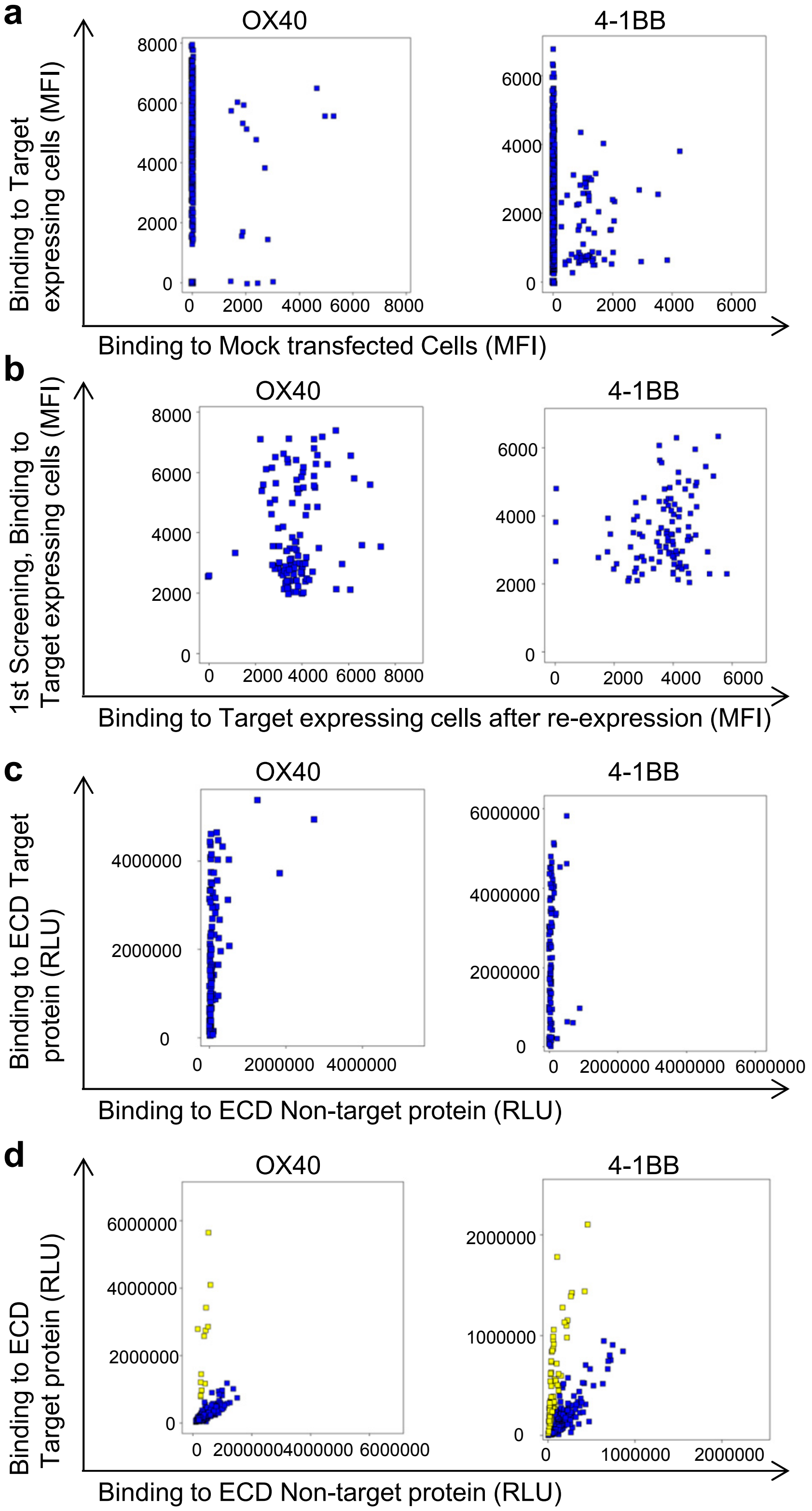

Supplementary figure 1: Binding-analysis of monospecific antibody fragments. (a) Screening in FMAT of individual soluble scFv showing the binding to target expressing cells versus binding to mock transfected cells. (b) Binding of unique clones to target expressing cells in the first screening versus their binding after re-expression. (c) Binding of clones in ELISA to coated recombinant target protein and a non-related protein carrying the same tag as the target (non-target). (d) Screening of VH-VLD Fab clones in ELISA for binding to target versus non-target protein. Yellow-marked clones were selected as binders and used for Sanger sequencing to identify unique sequences.

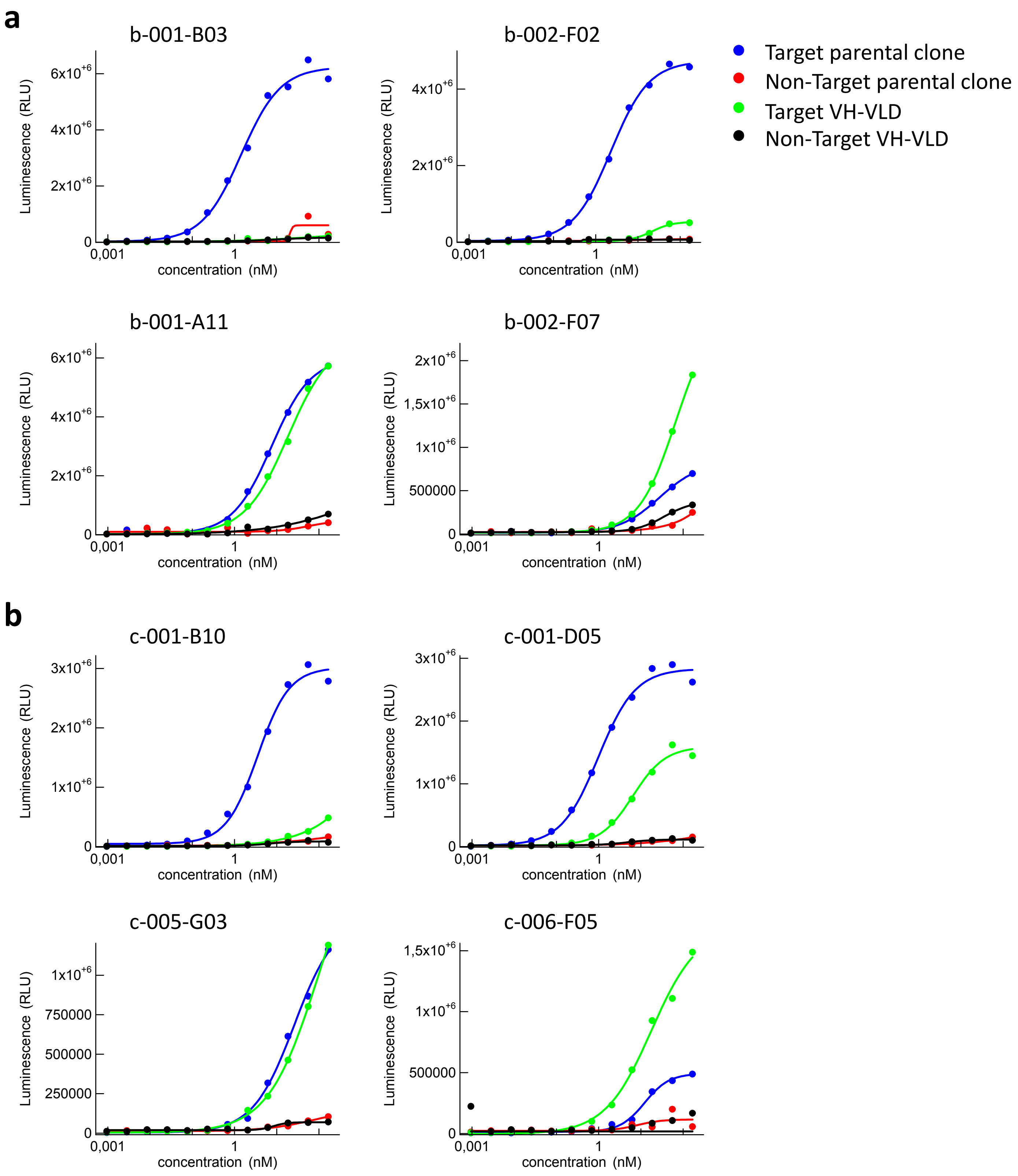

Supplementary figure 2: Dose response ELISA showing the binding of (a) anti-OX40 and (b) anti-4-1BB VH-VLD Fab clones, compared to their corresponding parental clones, in a Fab format, to the target protein and a non-target protein.

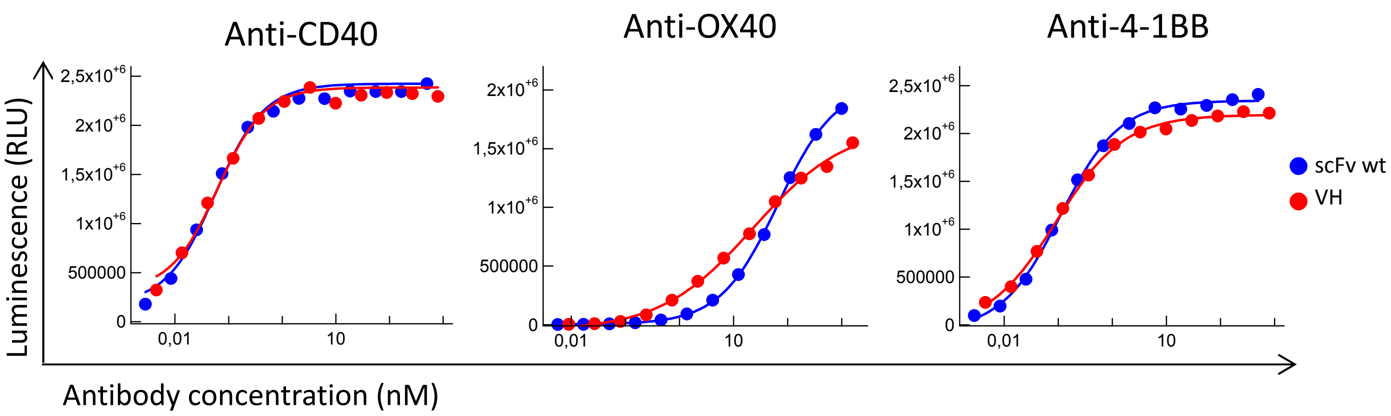

Supplementary figure 3: Dose response binding analysis of purified VHs domains alone compared to scFv containing the parental VH and VL combination.

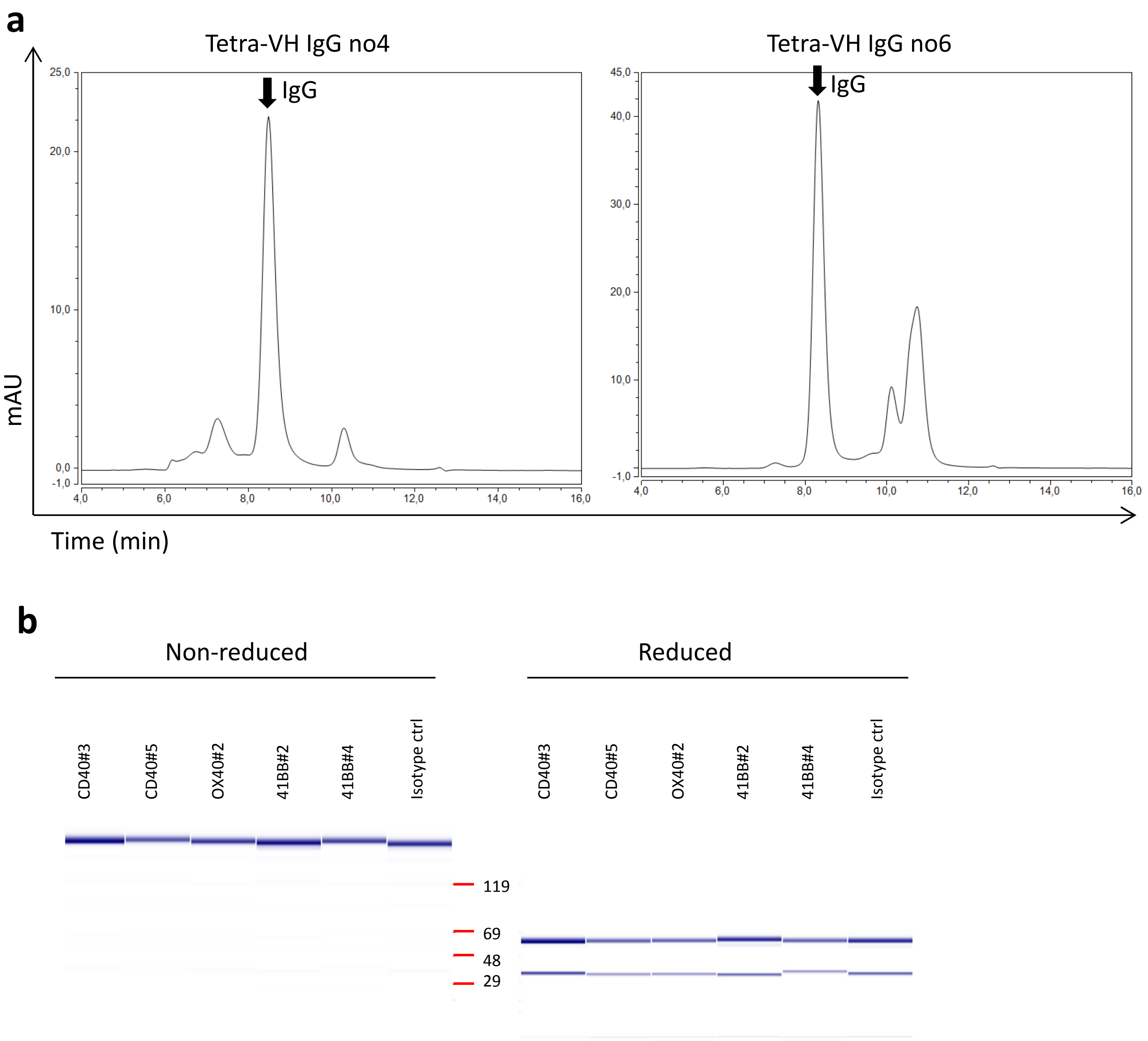

Supplementary figure 4: (a) Size exclusion chromatography (SEC) of antibodies purified on protein A before preparative SEC. (b) CE-SDS of tetra-VH IgGs under non-reduced and reduced conditions, a wild-type human IgG1 is included as control.

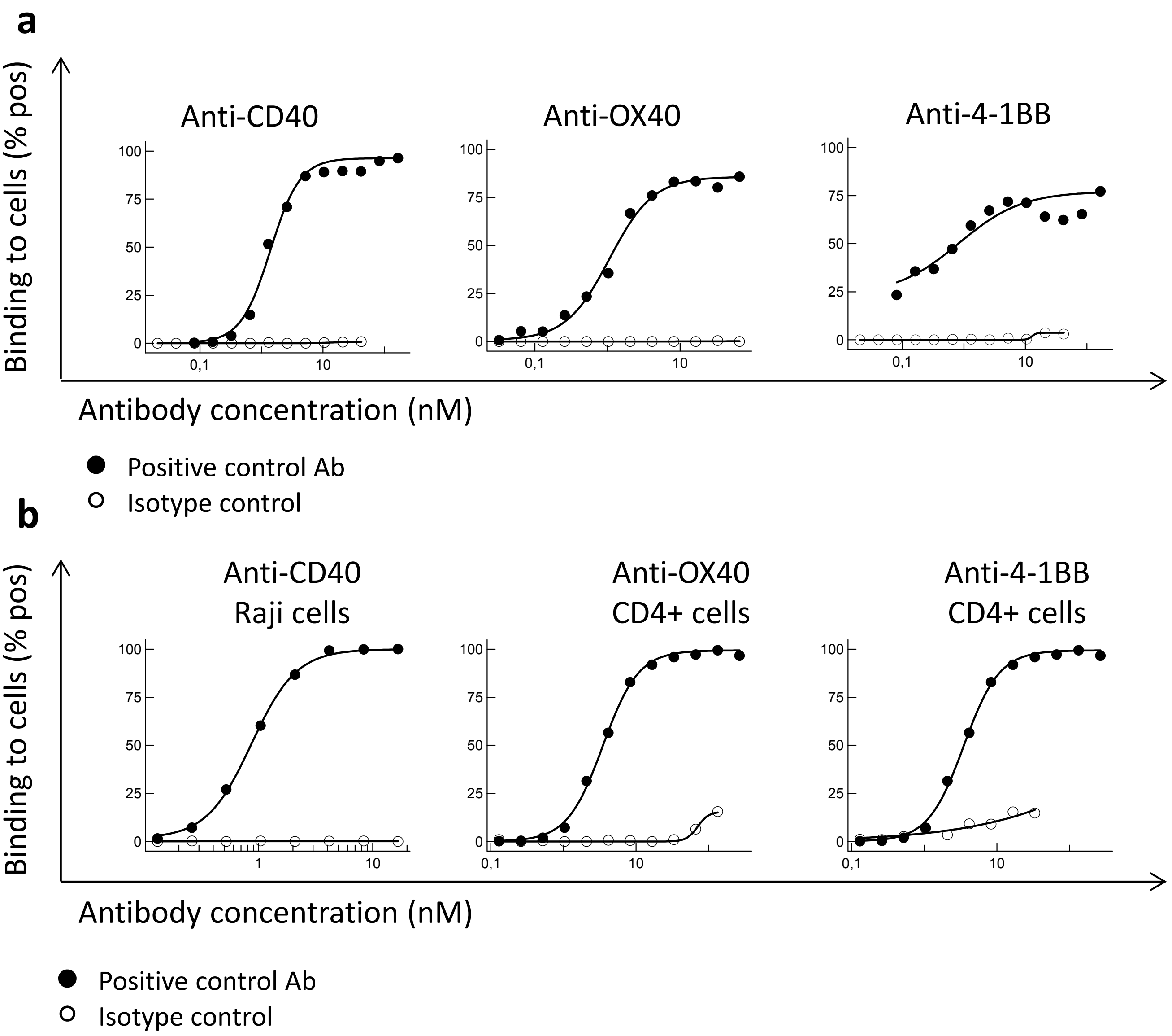

Supplementary figure 5: Binding-analysis of control antibodies to (a) transfected cells and (b) endogenously expressing cells in flow cytometry. An isotype antibody was included as negative control.

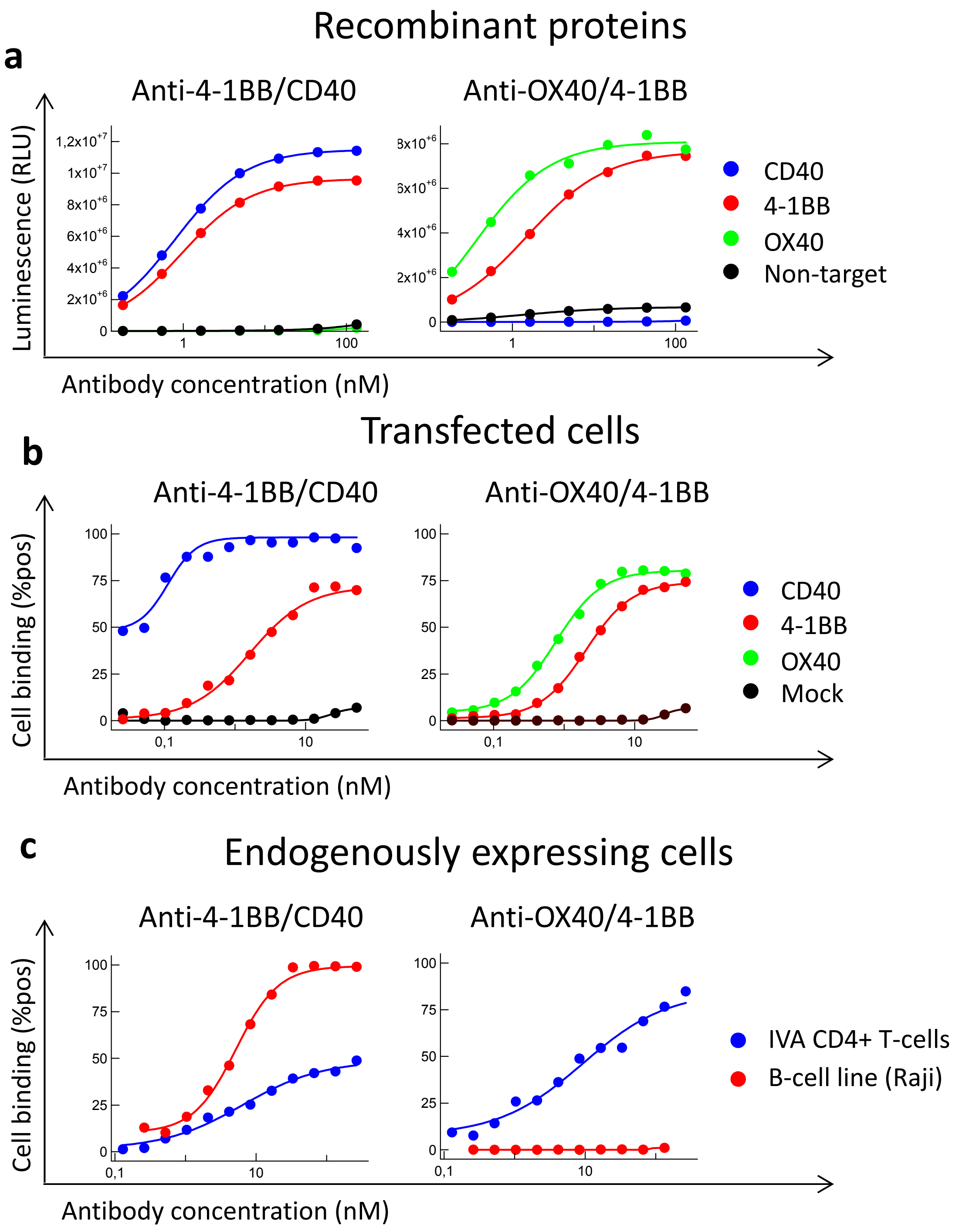

Supplementary figure 6: Binding-analysis of generated tetra-VH IgGs. Antibodies were evaluated for (a) binding to recombinant proteins in ELISA and for binding to (b) transfected cells or (c) endogenously expressing cells in flow cytometry.

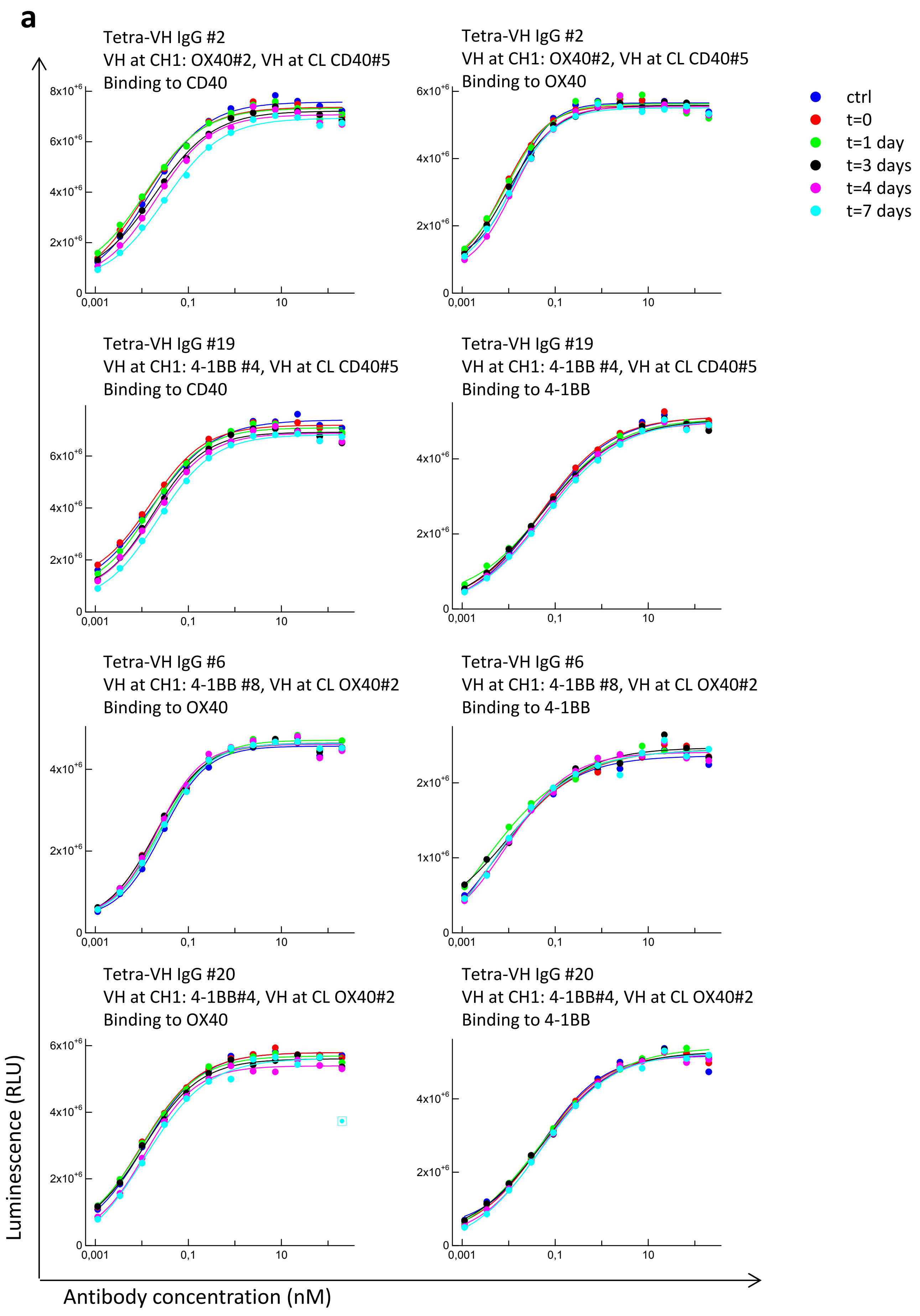

Supplementary figure 7

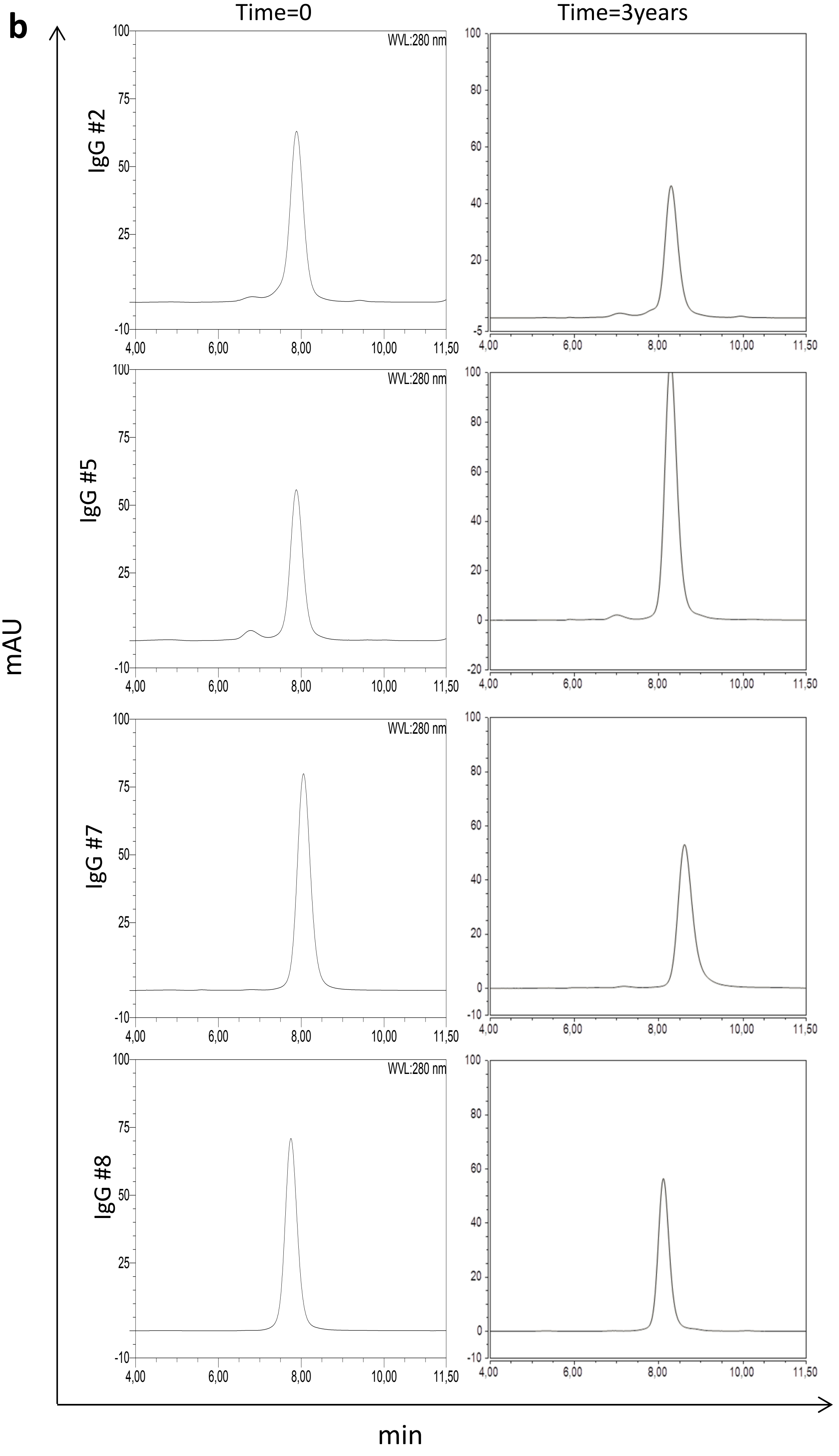

Supplementary figure 7 cont.

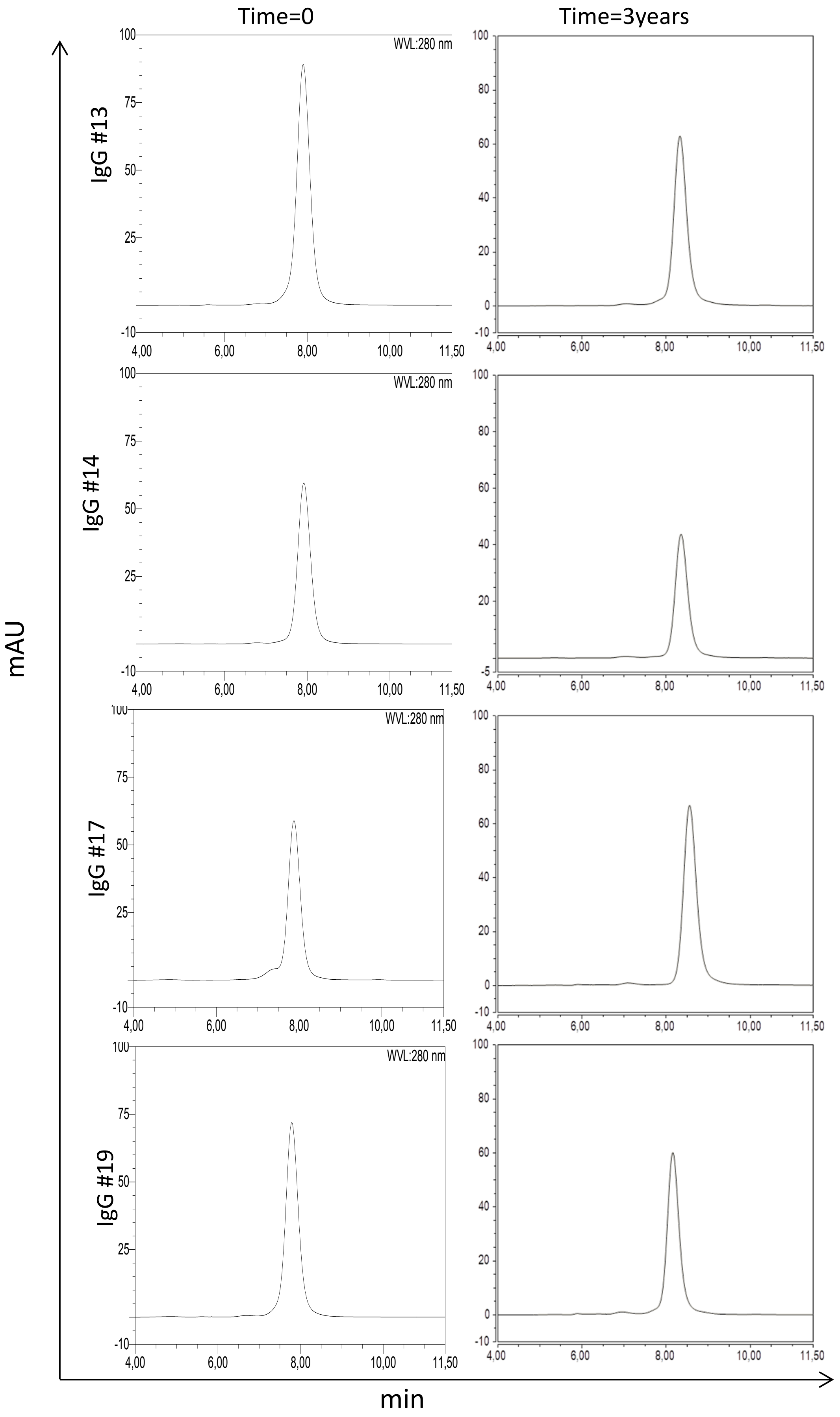

Supplementary figure 7 cont.

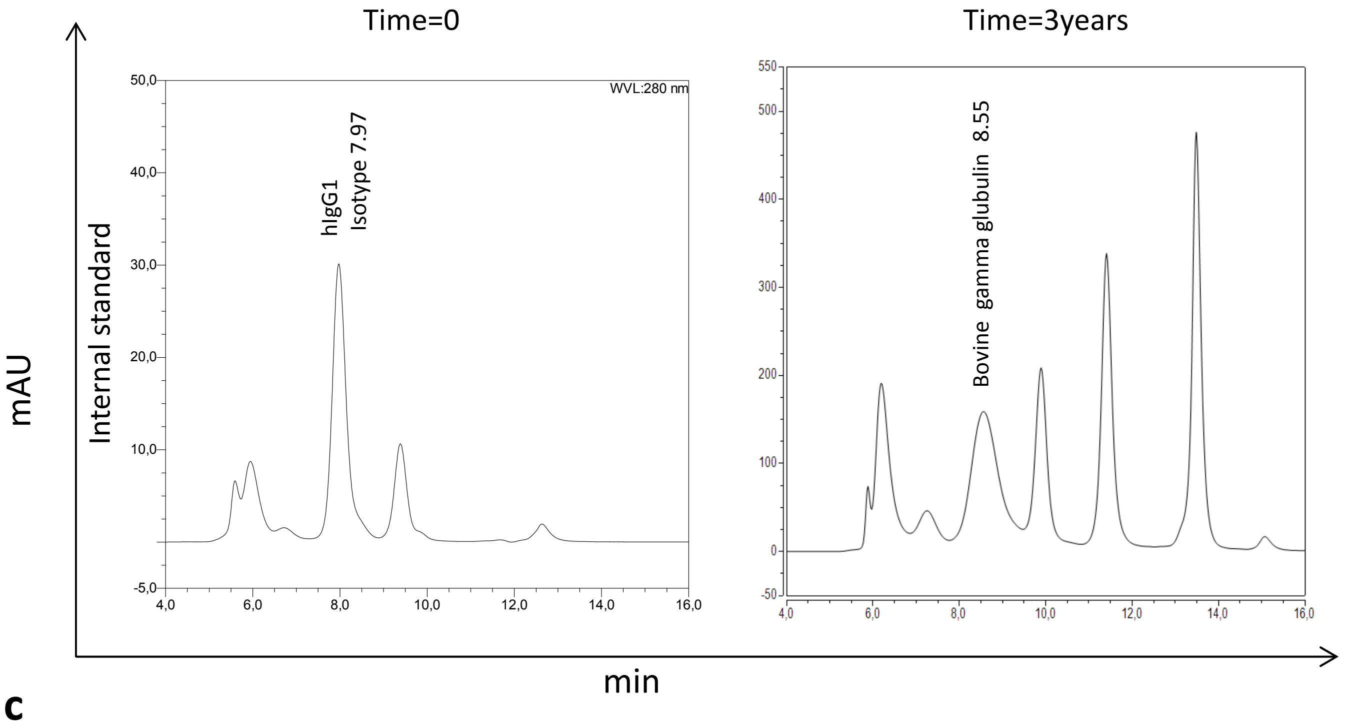

c

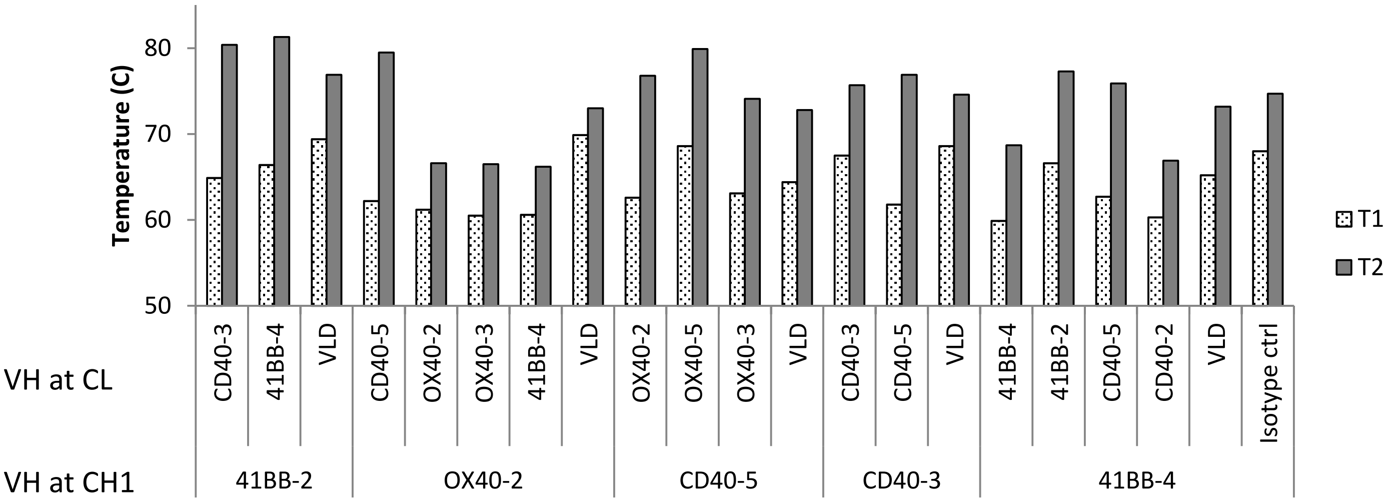

Supplementary figure 7: Stability of generated tetra-VH IgGs. (a) Binding of generated tetra-VH IgGs to coated antigen in ELISA after incubation in 50% human serum for 0, 1, 3, 4 and 7 days at +37°C. A fresh sample was diluted on the day of the binding analysis and included as control. Binding was detected using a HRP conjugated anti-human antibody and a luminescent substrate. (b) Size exclusion chromatography of purified tetra-VH IgGs after purification and after long term storage at +4°C. An internal standard containing Ig was included for comparison of retention times. (c) Antibody thermal stability measured with nanoDSF technology (Nano-temper technologies). Tetra-VH IgGs with VH linked to CH1, the normal position of VH, was combined with various VHs linked to CL. IgGs with a variable light dummy (VLD) chain were included for comparison.

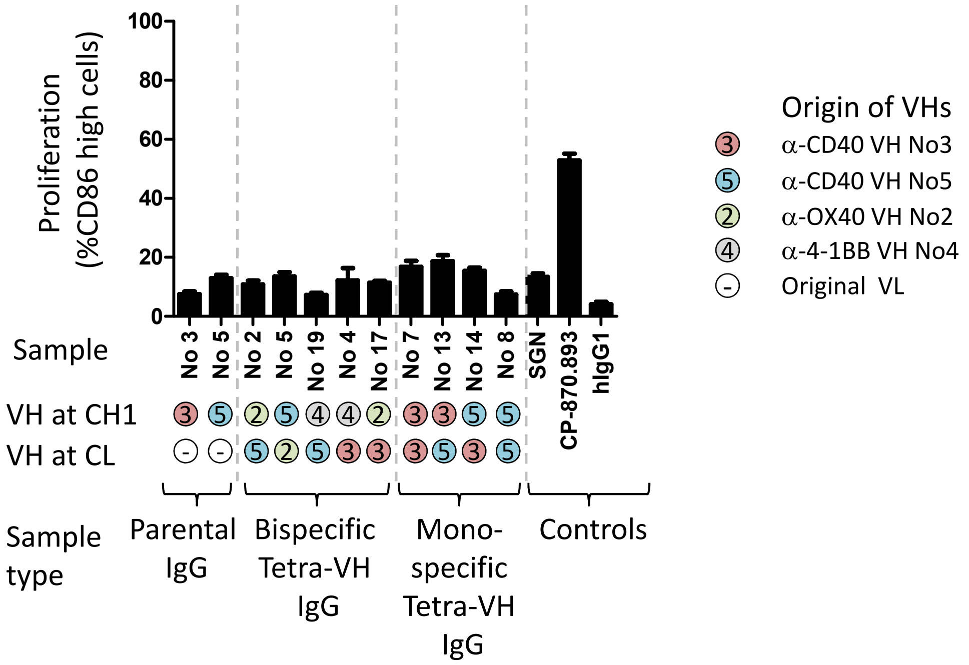

Supplementary figure 8: Evaluation of antibodies effect on B-cell proliferation. Wild-type parental IgGs, tetra-VH IgGs and control antibodies, without cross-linking, were analyzed for induction of B-cell proliferation on B-cells from healthy donors. Proliferation was measured as the percent of live CD19+ cells that were CD86 high. Samples were run as duplicates and data from 2-4 different donors were plotted as mean with SEM using GraphPad Prism.

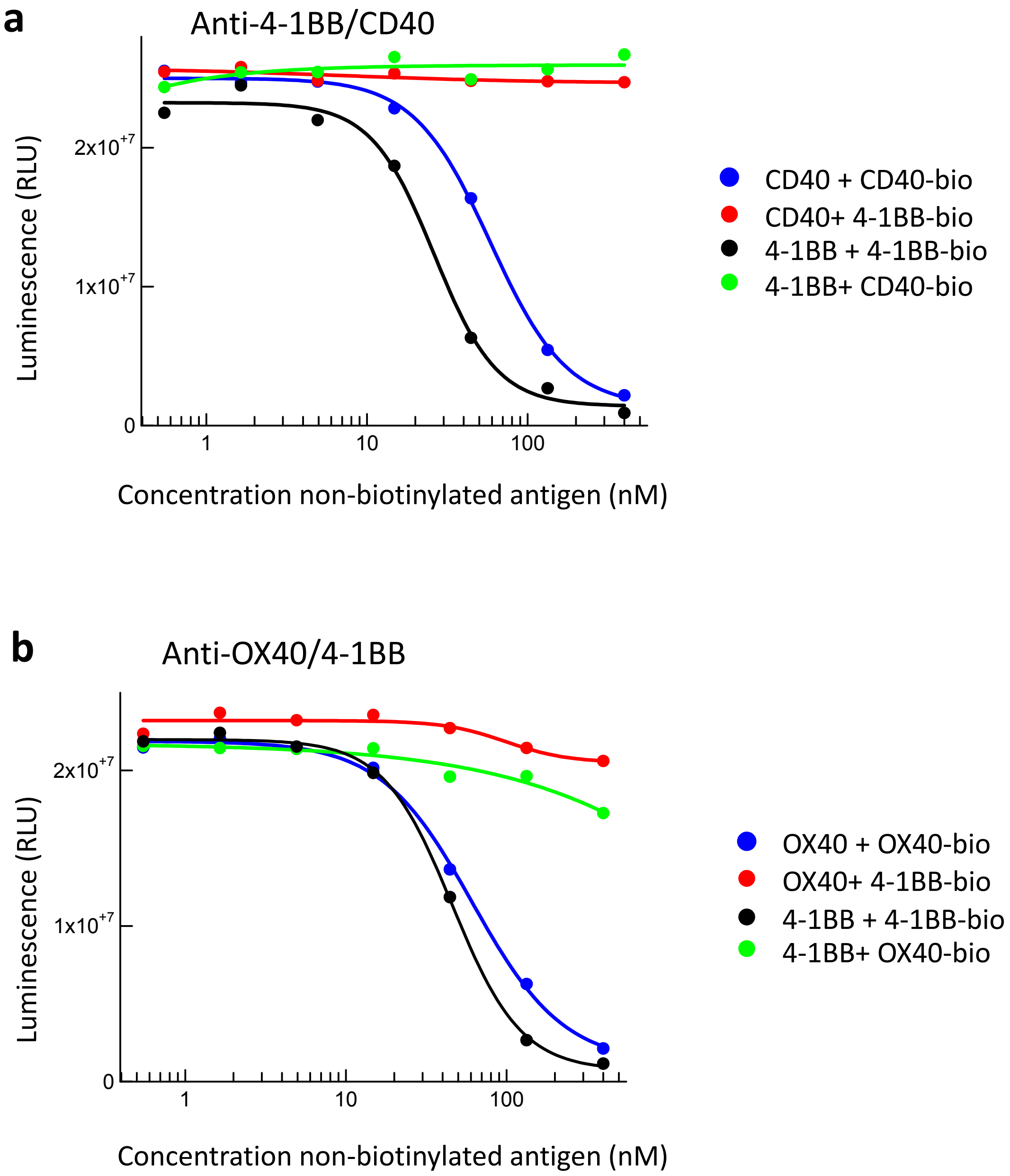

Supplementary figure 9: ELISA analysis of bispecific tetra-VH IgGs. (a) Analysis of an anti-4-1BB/CD40 tetra-VH IgG's binding to biotinylated 4-1BB or CD40 after blocking with non-biotinylated 4-1BB or CD40. (b) Analysis of an anti-OX40/4-1BB tetra-VH IgG's binding to biotinylated OX40 or 4-1BB after blocking with non-biotinylated OX40 or 4-1BB. The assay was run as described in 7a.

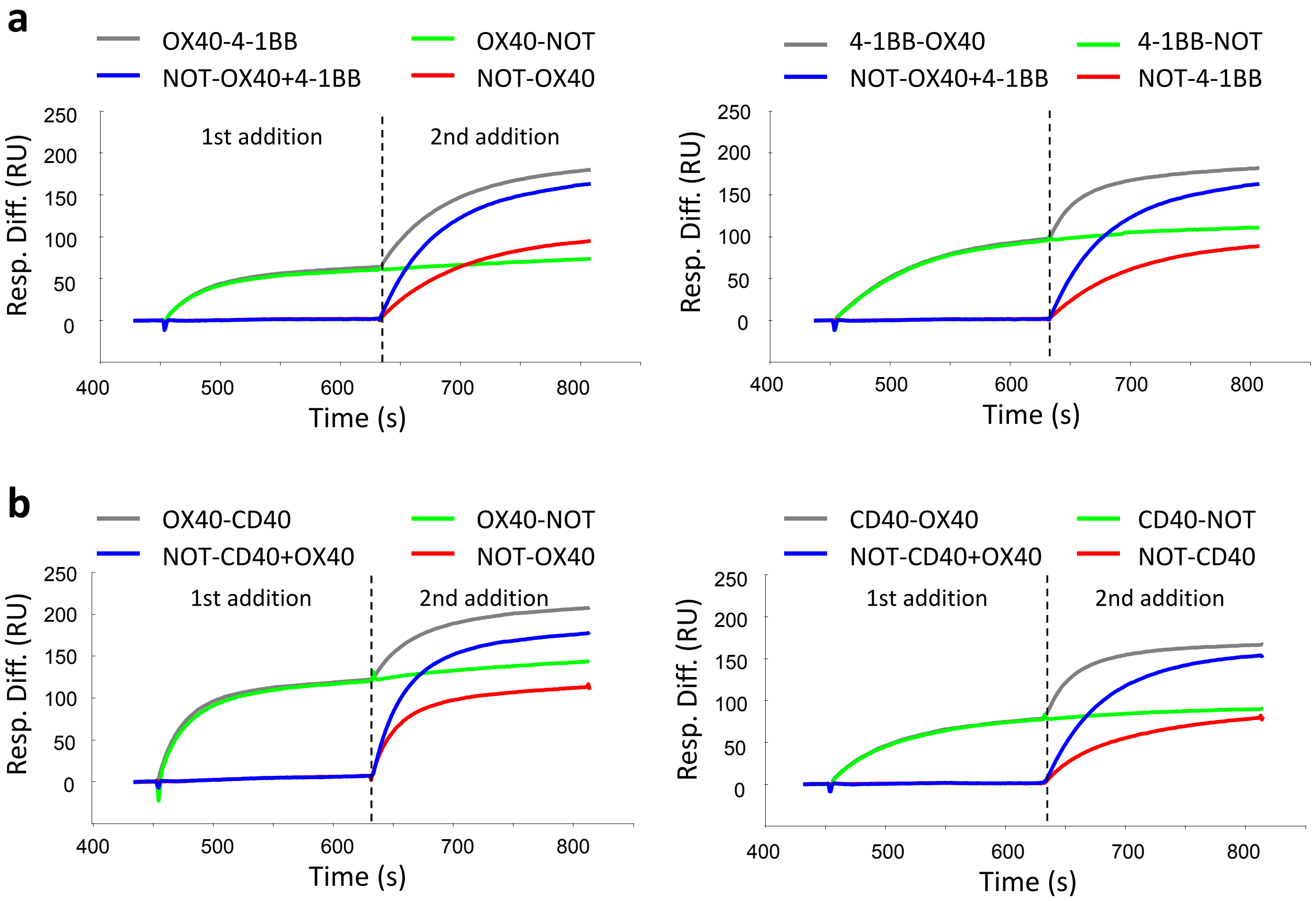

Supplementary figure 10: Binding-analysis of (a) anti-OX40/4-1BB and (b) anti-OX40/CD40 tetra-VH IgGs in Biacore. Antibodies were captured on an immobilized catcher antibody followed by addition of 800 nM of the first antigen to achieve binding saturation. The second antigen was then added at 800 nM, diluted in 800 nM of the first antigen, to avoid signal loss due to dissociation of the first antigen.

a

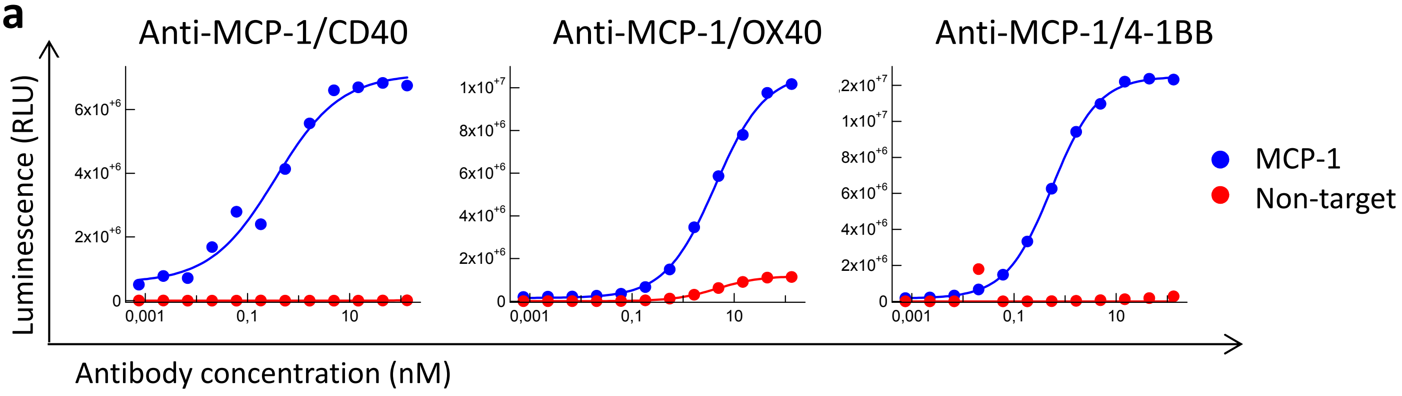

b

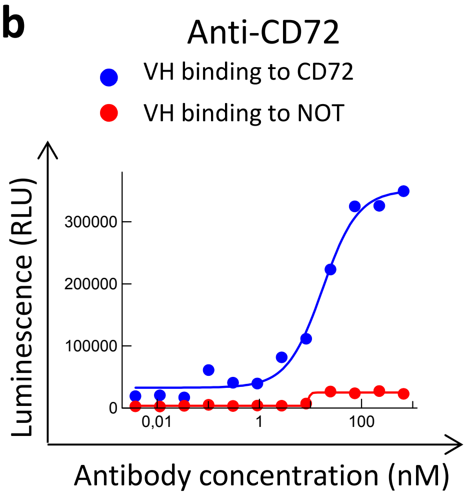

Supplementary figure 11: Binding of VHs to additional antigens. (a) Tetra-VH IgGs constructed with VHs binding to MCP1 or CD40, OX40 or 4-1BB, were analyzed for binding to coated MCP-1 in ELISA. (b) A purified VH domain, no VL, were analyzed for binding to CD72 in ELISA.

Supplementary table 1 No of clones in the various steps during construction of tetra-VH IgGs

	**CD40**	**OX40**	**4-1BB**
No of monospecific antibody fragments (scFv)	134	112	100
No of VH-binders (VH-VLD Fabs)	11	3	11
No of VHs tested in tetra-VH antibody format	8	3	8
No of VHs functional* in a tetra-VH antibody format	2	1	5

* Antibody can be expressed and VH retains binding

Supplementary table 2 EC50 values for tetra-VH IgGs binding to CD40 in ELISA directly after production of antibodies compared to after 3 years of storage.

**Sample**	**EC50 (nM) t= 0**	**EC50 (nM) t=3 years**
Anti-CD40 tetra-VH IgG No 2	1.1	1.0
Anti-CD40 tetra-VH IgG No 5	1.0	0.7
Anti-CD40 tetra-VH IgG No 7	0.6	0.4
Anti-CD40 tetra-VH IgG No 8	9.5	7.2
Anti-CD40 tetra-VH IgG No 13	0.8	0.4
Anti-CD40 tetra-VH IgG No 14	0.7	0.3
Anti-CD40 tetra-VH IgG No 17	12.4	4.1
Anti-CD40 tetra-VH IgG No 19	2.8	1.2
Pos ctrl-CP870.893	0.4	0.2
Pos ctrl-SGN-40	0.4	0.2

Supplementary Material and Methods

Preparation of CD40, OX40 and 4-1BB antigens

CHO-S cells (Thermo Fisher) were transfected with vectors encoding full-length CD40, 4-1BB and OX40 (Sino Biologicals HG10774-M-N, HG10481-G-N and HG10041-M-N) using FreeStyle MAX Reagent (Thermo Fisher) and used either 48h after transfection (4-1BB and OX40) or for stable cell line development (CD40). Stable cell lines were developed by addition of selection pressure, 600 µg/mL Hygromycin (Thermo Fisher) and limiting dilution of surviving transfected cells.

Genes encoding the extracellular domain (ECD), PCR amplified from full length constructs above, of CD40 (aa 1-193), OX40 (aa1-216) or 4-1BB (aa1-186), respectively, were ligated into an expression vector (in-house developed) containing a C-terminal 8x His-tag and produced in suspension-adapted HEK293 EBNA cells (ATCC, cat no. ATCC-CRL-10852). The proteins were purified from the culture medium (FreeStyle293 expression Medium, Thermo Fisher Scientific) with Ni-NTA Affinity Chromatography (ÄKTA purifier, GE Healthcare).

Isolation of monospecific antibody fragments

The phage display n-CoDeR scFv library[1] was used to generate CD40-, OX40- and 4-1BB- specific scFv. A selection scheme with 1) protein coated on polystyrene beads (Polysciences, cat no. 17175), 2) biotinylated proteins loaded on Streptavidin beads (Dynabeads M-280 Streptavidin, Thermo Fisher) and target-expressing cells was used for selection of such binders. Phages were recovered and amplified and the genes encoding the scFv used for conversion to soluble scFv as described previously[2].

Screening of monospecific antibody fragments

Individual scFv clones were analyzed for binding to target expressing CHO cells and mock transfected CHO cells in FMAT (Fluorometric Microvolume Assay Technology). 2500 cells/well in 40 µl DMEM media (Thermo Fisher cat no. 11330032) were added to FMAT plates (384 well, Thermo Fisher) followed by 10 µl/well of scFv supernatant and 20 µl/well of mouse anti-His (0.6 µg/ml, R&D Systems cat no. MAB050) and Allophycocyanin (APC)- labeled anti-mouse antibody (1.5 µg/ml,

Jackson ImmunoResearch cat no. 109-136-098). After 10 h incubation at room temperature, the plates were read in a 8200 Cellular detection system (Thermo Fisher Scientific). Unique clones binding specifically to the target cells (no detected binding to mock transfected cells and a signal above 2000 on target cells) were identified with Sanger sequencing (Eurofins Genomics).

Unique scFv clones were re-expressed, binding to cells confirmed in FMAT as described above, and further analyzed for binding to recombinant CD40, OX40 and 4-1BB proteins respectively, in Enzyme-linked immunosorbent assay (ELISA). The ECD of the target proteins were coated to ELISA plates overnight. After washing, scFv supernatants, 10 µl/well, diluted in block buffer 40 µl/well, (PBS (Invitrogen) with 0.05 % Tween 20 and 0.45 % fish gelatin (both from Sigma-Aldrich)) 40µl/well, were left to bind for 1h at room temperature. Bound scFv was detected with an alkaline phosphate (AP)- labeled anti-FLAG M2 antibody (Sigma Aldrich cat no. A9469) and a luminescent substrate (CDP Star Emerald II, Thermo Fisher cat no. T2216) and plates were read in a plate reader (Tecan Ultra).

Isolation of monospecific VH binders

Genes encoding VHs from all unique scFv were amplified by PCR, pooled and ligated into a Fab expression vector (in-house developed) containing a VL dummy gene, composed of the light chain variable gene IgLV1-47*01 (accession no Z22189) rearranged to the light chain joining gene fragment IgLJ3*02 (accession no D87023). Chemically competent E. coli Top10 were transformed and individual clones picked and used for production of soluble VH-VLD Fabs. These Fabs were analyzed for binding to coated proteins in ELISA as described above for scFv. Clones specifically binding to CD40, OX40 and 4-1BB respectively were sequenced with Sanger (Eurofins Genomics) for rediscovery of VHs binding also in combination with this irrelevant VL domain.

Unique VH-VLD binding Fabs were purified from periplasm of E. coli as described previously[2]. Purified Fabs were titrated for binding their corresponding target antigen in a dose response ELISA. Target protein and non-target protein were coated to ELISA plates overnight at +4°C. Purified Fabs were serially diluted 1:3, starting at 200 nM, and added to the coated and washed ELISA plates. Bound Fabs were detected as described above.

Construction of tetra-VH IgG antibodies

The genes encoding the VH domains of VH-VLD binding Fabs were PCR amplified from the original scFv vector and ligated into two expression vectors (proprietary, in-house developed) to enable production of tetra-VH human IgG antibodies. The expression vectors contain a CMV-promoter, a BGH poly A signal and an ampicillin resistance gene. In addition, the heavy chain vector contains the gene encoding the antibody heavy chain IgG1 constant region and the light chain vector contains the gene encoding the antibody lambda light chain constant domain. Two independent VHs (binding the same or different target antigens) were used. One VH encoding gene is inserted in the vector containing the gene encoding the constant heavy chain (normal position), whereas the other VH is inserted in the vector containing a gene encoding the lambda light chain constant domain (CL), thereby replacing VL in the encoded product. The vectors were prepared from E. coli according to manufacturer's instructions (Qiagen miniprep kit cat no. 27104) and used for transient transfection of suspension-adapted HEK 293 EBNA (Thermo Fisher Scientific) with polyethylenimine (PEI) (Polyscience Inc cat no. 23966). The cells were incubated for 4h, +37°C, 8% CO_2, 300rpm before addition of feed, UltraPep Soy (Sheffield Bio-Science). After 6 days, cell supernatants were harvested and IgG purified on Mabselect (GE Healthcare) followed by preparative size-exclusion chromatography (SEC) to remove unpaired light chains. After purification, antibodies were analyzed by analytical SEC (Ultimate3000, Thermo Fisher) and this was repeated after long term storage at +4°C. Antibodies were also analyzed in CE-SDS (Capillary electrophoresis sodium dodecyl sulfate) (LabChip GX II, Perkin Elmer) according to the manufacturer´s instructions (HT Protein Express LabChip Kit, Version 2 LabChip GXII User Guide) under both reduced and non-reduced conditions. A wild-type human IgG1 was included as control for comparison.

Binding analysis of tetra-VH IgG antibodies

The binding of purified tetra-VH IgG antibodies was confirmed in ELISA and flow cytometry. For ELISA, target proteins were coated to ELISA plates over night at +4°C. The next day antibodies were serially diluted 1:3 starting at 133 nM and left to bind the washed ELISA plate for 1h at room temperature. Binding was detected using a horseradish peroxidase (HRP) conjugated anti-human-Fc

antibody (Jackson ImmunoResearch cat no. 709-036-098), followed by a luminescent substrate (SuperSignal ELISA Pico Chemiluminescent Substrate , Thermo Fisher, cat no. 37070) and reading in a plate reader (Tecan Ultra, Tecan).

For cell binding analysis, transfected CHO cells, in vitro-activated T-cells, as described below, or a B-cell line (Raji ATCC cat no. CCL-86) were added at 50 000 cells/well. Target expression was confirmed with mIgG2b anti-CD40-APC (R&D Systems cat no. FAB6321A), rat IgG2a anti-OX40-APC (R&D Systems cat no. FAB3388A) and mIgG2b anti-4-1BB-APC (BioLegend cat no 309809) with corresponding isotype controls, mouse IgG2B-APC (BD cat no. 555745) and rat IgG2A-APC (BD cat no. 560720). IgG preparations were serially diluted 1:3 starting at 133 nM and left to bind the cells for 1 h at +4°C. After washing, bound IgG was detected with anti-human F(ab)'2-APC (Jackson ImmunoResearch, cat no. 109-136-098) antibody by flow cytometry (FACS Verse, BD Biosciences). For in vitro activation of T-cells, CD4+ T-cells were purified from peripheral blood mononuclear cells (PBMC) by negative selection (human CD4+ T-cell isolation kit, Miltenyi, cat no. 130-096-533) and activated with 50 ng/ml of IL2 (R&D Systems) and CD3/CD28 beads (Dynabeads, Thermo Fisher Scientific cat no. 11161D) at a 1:1 cell:bead ratio. Cells were incubated 2-5 days at +37°C, 8% CO_2 before analysis.

B-cell proliferation assay

PBMC from healthy donors were prepared from buffy coats by density gradient centrifugation (Ficoll Paque PLUS, GE Healthcare). B-cells were purified by negative selection according to manufacturer's instructions (pan B cell isolation kit, Miltenyi, cat no. 130-091-151) and added to plates at 100 000 cells/well in media containing 10 ng/ml of IL4 (R&D Systems cat no. 204-IL). Antibodies, at 50 nM, were analyzed with or without crosslinking and incubated with cells in duplicates. When stated antibodies were cross-linked with F(ab)'2-anti-human-Fc (Jackson ImmunoResearch, cat no 109-006-098) at a molar ratio of 1.5:1. After 5 days incubation at +37°C, 8% CO2, cells were stained with anti-CD19-BV421 (BD Biosciences, cat no. 562440), anti-CD86-APC (BD Biosciences, cat no. 555660) and a live dead marker (Thermo Fisher, propidium iodide, alternatively eBioScience, cat no. 65-0865-14) and analyzed by flow cytometry (FACS Verse, BD Biosciences). B-cells purified from 2-4 donors

were used to evaluate the activity of the tetra-VH IgGs. B-cell proliferation was analyzed by the fraction of live CD19+ and CD86 high cells. For dose response experiments, all antibodies were cross-linked and values for the isotype control was subtracted from the value of the analyzed IgGs.

ELISA analysis of tetra-VH IgGs for evaluation of valency

The tetra-VH IgGs ability to bind two antigens simultaneously was assessed in ELISA, schematically outlined in Figure 7a. IgG at 16 nM was captured on a coated (7.5 µg/ml) goat anti-human-Fc antibody (Jackson ImmunoResearch, cat no. 109-005-008) microtiter plates. After washing, non-biotinylated antigen, diluted to 400 nM, was titrated 1:3 and added to the wells, followed by biotinylated antigen addition at a fixed concentration resulting in a high, but titratable, signal. After washing, bound biotinylated antigen was detected using HRP-labeled streptavidin (Jackson ImmunoResearch, cat no. 016-030-084) followed by a luminescent substrate (SuperSignal ELISA Pico Chemiluminescent Substrate, Thermo Fisher, cat no. 37070) and reading in a plate reader (Tecan Ultra, Tecan).

A bridging ELISA was performed to further investigate if each arm of the tetra-VH IgGs could bind two antigens simultaneously. VH-VH Fabs (produced as described for VH-VLD Fabs above) were left to bind the first antigen coated to an ELISA plate. The second antigen, in a biotinylated version, was diluted to 400 nM and titrated 1:3 and added to the plate with bound VH-VH Fab. After washing, a second antigen was added at different concentrations in a biotinylated version. Thereafter, bound biotinylated antigen was detected using HRP-labeled streptavidin (Jackson ImmunoResearch, cat no. 016-030-084) and a luminescent substrate (SuperSignal ELISA Pico Chemiluminescent Substrate) followed by reading in a plate reader (Tecan Ultra, Tecan). A non-target protein and the coated antigen added in a biotinylated form were included as controls.

References

1 Soderlind, E. *et al.* Recombining germline-derived CDR sequences for creating diverse single-framework antibody libraries. *Nat Biotechnol* **18**, 852-856 (2000).

2 Ljungars, A. *et al.* Deep Mining of Complex Antibody Phage Pools Generated by Cell Panning Enables Discovery of Rare Antibodies Binding New Targets and Epitopes. *Frontiers in Pharmacology* **10**, doi:10.3389/fphar.2019.00847 (2019).
